# Engineering of Transmembrane Alkane Monooxygenases to Improve a Key Reaction Step in the Synthesis of Polymer Precursor Tulipalin A

**DOI:** 10.1002/anie.202503464

**Published:** 2025-05-16

**Authors:** Andrea Nigl, Veronica Delsoglio, Lucija Sovic, Marina Grgić, Lenny Malihan‐Yap, Kamela Myrtollari, Jelena Spasic, Margit Winkler, Gustav Oberdorfer, Andreas Taden, Iva Anić, Robert Kourist

**Affiliations:** ^1^ Institute of Molecular Biotechnology, Graz University of Technology Petersgasse 14 Graz 8010 Austria; ^2^ acib GmbH (Austrian Centre of Industrial Biotechnology) Petersgasse 14 Graz 8010 Austria; ^3^ Institute of Biochemistry, Graz University of Technology Petersgasse 12/2 Graz 8010 Austria; ^4^ Henkel AG & Co. KGaA Henkelstraße 67 40589 Düsseldorf Germany; ^5^ BioTechMed‐Graz Mozartgasse 12/II Graz 8010 Austria

**Keywords:** Alkane monooxygenase, Polymers, Protein engineering, Synthetic pathways, Tulipalin A

## Abstract

The α‐methylene‐γ‐butyrolactone tulipalin A, a defense compound found in tulips, can polymerize via addition at the vinyl group or via ring‐opening polymerization, making it a highly promising monomer for bio‐based polymers. Since the biosynthesis of tulipalin A in plants remains elusive, we propose an alternative pathway for its synthesis starting from the terpenoid intermediate isoprenyl acetate. While fungal unspecific peroxygenases showed a preference for the unwanted epoxidation of the *exo‐*olefin group, bacterial alkane monooxygenases were selective for terminal hydroxylation. By combining protein engineering based on de novo structure prediction of the membrane enzymes with cell engineering, the specific activity was increased 6‐fold to 1.83 U g_cdw_^−1^. Oxidation of the formed allylic alcohol by a three‐enzyme cascade and subsequent lactonization yielded tulipalin A. Our results demonstrate the feasibility of producing the polymer precursor tulipalin A from terpenoid intermediates and provide a solid foundation for future metabolic engineering endeavors.

## Graphical abstract

A biosynthesis route for the polymer precursor tulipalin A starting from isoprenyl acetate is proposed. The alkane monooxygenase AlkB catalyzes the chemoselective terminal hydroxylation of isoprenyl acetate. Rational enzyme engineering further increased the activity of the membrane enzyme. An alcohol and aldehyde dehydrogenase pair enabled complete oxidation to the carboxylic acid, thus paving the way to biotechnological tulipalin A production.

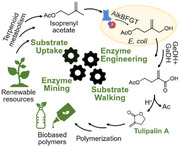

Tulipalin A (**1e**, α‐methylene‐γ‐butyrolactone) is a secondary metabolite found in plants among the Alstroemeriaceae and Liliaceae families. It exhibits strong antimicrobial and insecticidal properties with a broad target spectrum.^[^ ^1^, ^2^, ^3^, ^4^ ^]^ For instance, **1e** has been shown to be an effective pesticide against various insect pests.^[^ ^4^ ^]^ In addition to its interesting biological features, **1e** combines two functional moieties accessible for polymerization within one molecule — a lactone and a vinyl group. Its *exo*‐methylene group makes **1e** a potential substitute for the petrol‐based monomer methyl methacrylate (MMA), an important component of reactive adhesives, coatings, and sealants.^[^ ^5^, ^6^, ^7^ ^]^ The homopolymer of **1e** is characterized by a high glass transition temperature and shows good heat, scratch, and solvent resistance.^[^ ^5^, ^6^ ^]^ The lactone can also undergo ring‐opening polymerization (ROP), resulting in unsaturated (biodegradable) polyesters that can be further cross‐linked to form polymeric networks.^[^ ^5^, ^6^, ^8^, ^9^, ^10^ ^]^ Such polymeric networks based on **1e** can serve as superabsorbent hydrogels for agricultural or biomedical applications.^[^ ^11^, ^12^, ^13^ ^]^ The versatility of tulipalin A makes it an interesting building block for a wide range of high‐performance polymers.^[^ ^5^, ^9^ ^]^ While **1e** can be extracted from tulips, isolating a bulk chemical from plant material is not economically feasible. Even though the final step of tulipalin A synthesis *in planta* is well understood (Scheme 1a),^[^ ^3^, ^14^, ^15^, ^16^ ^]^ the complete biosynthesis pathway remains elusive, hindering the development of biotechnological processes based on the natural pathway.^[^ ^14^, ^17^, ^18^, ^19^ ^]^

**Scheme 1:**
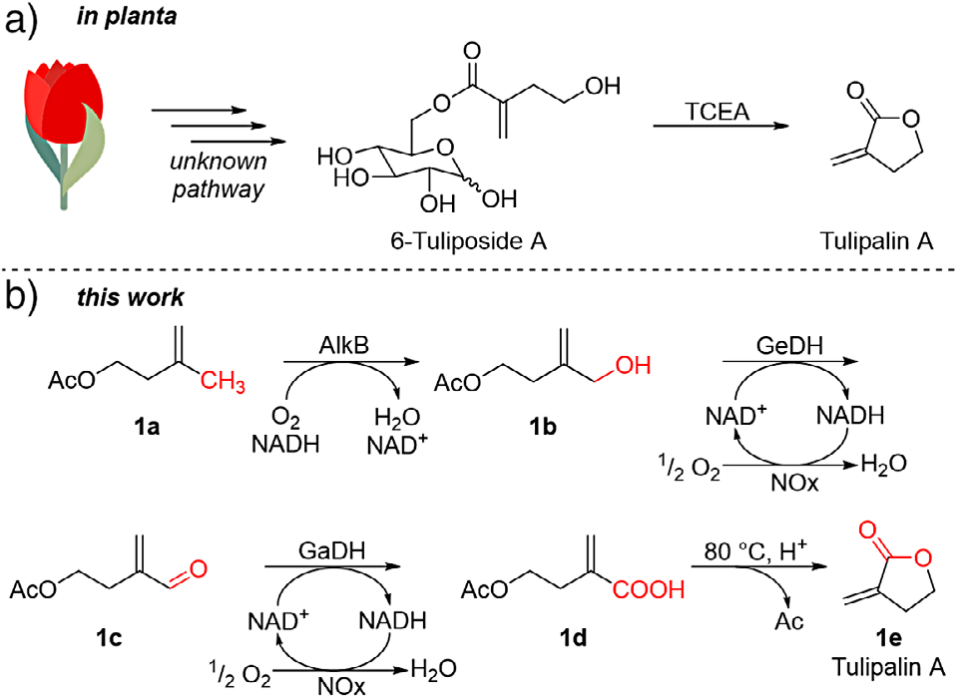
a) *In planta* synthesis of the storage form tuliposide A remains unknown. Upon plant cell damage, tulipalin A is released by the tuliposide A converting enzyme (TCEA). b) This work proposes an artificial pathway toward tulipalin A **1e**, deviating from a terpenoid precursor, and starting with whole‐cell hydroxylation of isoprenyl acetate **1a**, combined with a cell‐free multi‐enzyme cascade reaction to **1d**, and lactonization under acidic conditions yields **1e**.

Here, we propose the synthesis of **1e** starting from isoprenyl acetate **1a** (Scheme 1b), which can be readily produced in engineered microorganisms from the hemiterpenoid isoprenol with titers of up to 28 g L^−1^.^[^ ^20^, ^21^, ^22^, ^23^, ^24^ ^]^ The acetate protects the C1‐OH moiety of the metabolic intermediate isoprenol from oxidation in the later phase of the pathway. Selective hydroxylation of **1a** at the C4‐position yields the alcohol **1b**, which is fully oxidized to the carboxylic acid **1d** (via aldehyde **1c**) and further lactonized to **1e** by acidification.^[^ ^18^, ^25^ ^]^ The crucial step in this synthetic route is the regioselective hydroxylation of isoprenyl acetate **1a** to 4‐acetoxy‐2‐methylene‐butan‐1‐ol **1b**. It requires a chemo‐ and regio‐selective oxygenase that neither catalyzes the epoxidation of the *exo*‐double bond nor the internal hydroxylation of **1a**.

Since the discovery of unspecific peroxygenases (UPO) from *Agrocybe aegerita* (*Aae*UPO), these highly active and stable enzymes have attracted increasing attention for cell‐free C‐H‐oxyfunctionalization reactions, making them promising candidates for the desired hydroxylation of **1a**.^[^ ^26^, ^27^, ^28^, ^29^ ^]^ Investigation of a commercial enzyme panel of 77 UPOs revealed that *Aae*UPO and 71 other UPOs indeed accept **1a** as substrate. Unfortunately, the formation of **1b** was not observed for any of the enzymes tested. Instead, the epoxide **1f** (Figure 1a, Figure S3, Table S10) was formed.^[^ ^30^, ^31^ ^]^ Due to the indicated preference of UPOs for epoxidation, we found it unlikely to identify a UPO selective for C4‐hydroxylation. A complete chemoselectivity switch from epoxidation to hydroxylation by enzyme engineering was deemed exceedingly difficult.

**Figure 1:**
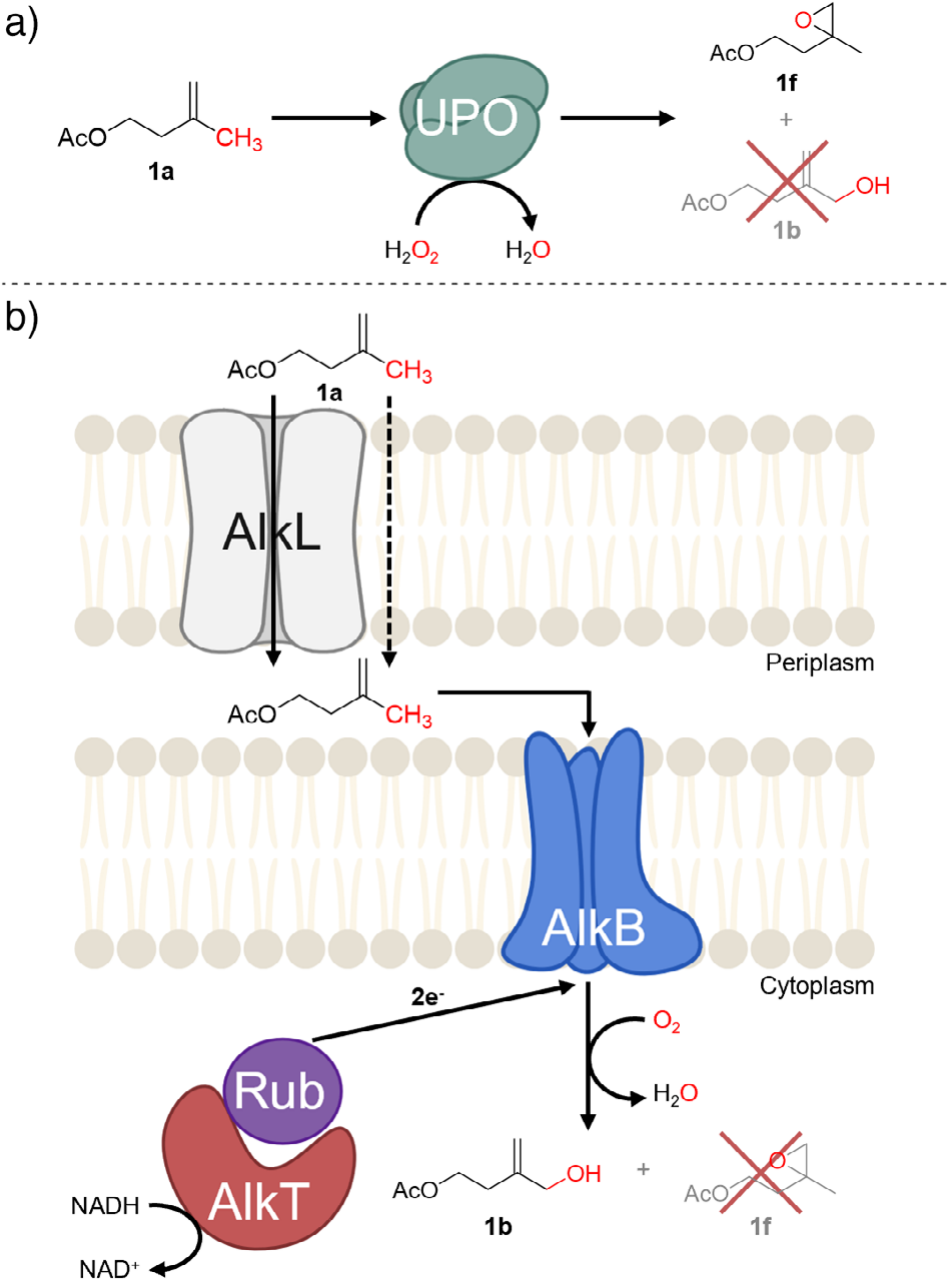
Conversion of isoprenyl acetate **1a** by a) heme‐dependent UPOs gives rise to the epoxide **1f**, while the reaction with b) the AlkBFGT(L) yields the desired terminal alcohol **1b** (created using BioRender.com).

Hence, we investigated a different type of monooxygenases (MO) for the selective hydroxylation of **1a**: The transmembrane, diiron, non‐heme alkane monooxygenase AlkB from *Pseudomonas putida* GPo1 (PpGPo1AlkB) has been reported to catalyze the hydroxylation of isobutene to 2‐methyl‐2‐propen‐1‐ol while leaving the olefin group unreacted.^[^ ^32^ ^]^ We hypothesized that AlkB should be able to terminally hydroxylate the structurally similar **1a** without undesired oxidation of the double bond or internal hydroxylation. In nature, AlkB is part of the *alk*‐operon and catalyzes the initial hydroxylation in alkane‐degrading pathways present in many soil and marine bacteria.^[^ ^33^, ^34^ ^]^

The remarkable selectivity of AlkB for ω‐hydroxylation of linear alkanes is a striking difference from the widely applied CYP450s that often hydroxylate also the ω‐1 and ω‐2 positions of aliphatic compounds.^[^ ^35^, ^36^, ^37^, ^38^, ^39^, ^40^ ^]^ PpGPo1AlkB hydroxylates the acetate esters of various fatty alcohols without acting on the acetate group,^[^ ^41^ ^]^ and was successfully applied for the ω‐hydroxylation of fatty acid methyl esters at an industrial scale.^[^ ^42^, ^43^, ^44^ ^]^

Here, the genes of the AlkBFGT system from *P. putida* GPo1 were recombinantly expressed in *Escherichia coli* BL21 (DE3) employing the natural alkane‐inducible *alkB* promoter.^[^ ^38^, ^45^ ^]^ The gas chromatography‐mass spectrometry (GC‐MS) analysis of whole‐cell biotransformations with **1a** showed no formation of **1f**. However, a peak with a mass spectrum matching the desired product **1b** was observed (Figure S5). The product was extracted and purified from a 20 mL scale reaction and analyzed via nuclear magnetic resonance (NMR) spectroscopy, confirming the formation of **1b**.^[^ ^46^ ^]^ Besides chemo‐ and regioselectivity, the specific activity of the biocatalyst is a decisive parameter for industrial application. The activity of *E. coli* PpGPo1AlkBFGT was 0.28 U g_cdw_^−1^ (Figure 2a). While this allowed the identification of **1b**, the activity with the sterically hindered **1a** was significantly lower compared to the natural substrate *n‐*octane (4.43 U g_cdw_^−1^; Figure S8) and deemed to be a bottleneck for the application in the artificial biosynthesis route. To increase the product formation rate in the whole‐cell biotransformations, three different strategies were employed: a) exploration of homologous enzymes for their ability to hydroxylate **1a**; b) the use of the outer‐membrane transporter AlkL to facilitate cellular uptake, and c) semi‐rational engineering of PpGPo1AlkB based on literature data,^[^ ^47^, ^48^ ^]^ *de novo* structure prediction,^[^ ^49^ ^]^ and substrate docking.^[^ ^38^, ^43^ ^]^

**Figure 2:**
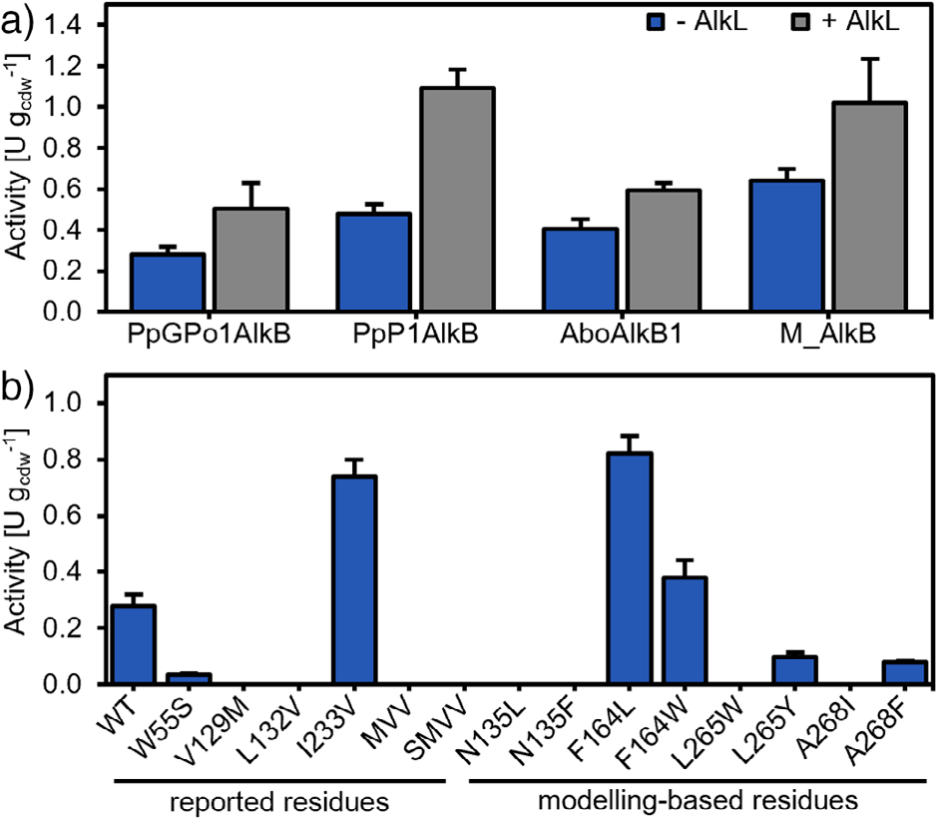
Whole‐cell activity toward isoprenyl acetate **1a** of a) wildtype (WT) AlkB homologs from different organisms without (blue) and with AlkL (grey) present; and b) screening of rationally designed PpGPo1AlkB variants (without AlkL) guided by reported mutations and docking of **1a** into the AF2‐structure of PpGPo1AlkB. Data represented as arithmetic mean and standard deviation indicated by error bars (*n = 3*).

Four homologous MOs with varying sequence identities to PpGPo1AlkB were selected. AlkB from *P. putida* P1 (PpP1AlkB; 90% sequence identity) and *Alcanivorax borkumensis* (AboAlkB1; 77%) were described in the literature to have a similar substrate scope as PpGPo1AlkB and were shown to accept electrons from the PpGPo1 rubredoxins.^[^ ^50^, ^51^ ^]^ The alkane MO from *Acinetobacter baylyi* (AbaAlkB; 41%) is known to act on medium to long‐chain alkanes.^[^ ^52^ ^]^ The putative alkane MO sequence from *Marinobacter* sp. (M_AlkB; 78%) originates from a marine metagenome study.^[^ ^53^ ^]^ The four genes were cloned into the same expression vector, replacing *PpGPo1alkB* while retaining the genes of the redox partners from *P. putida *GPo1. The functionality of the enzymes was tested using *n‐*octane as a reference substrate (Figure S8A). As shown in Figure 2a PpP1AlkB and AboAlkB1 were similarly active toward **1a**. AbaAlkB was poorly produced (data not shown) and no activity was observed, hence, it was not investigated further. M_AlkB was about 2‐fold more active toward **1a** and *n*‐octane. Epoxidation of **1a** was observed with neither of the tested alkane MOs. This underlined the outstanding chemoselectivity of these enzymes and confirmed our initial expectation based on the reactivity of PpGPo1AlkB toward isobutene.^[^ ^32^ ^]^

The facilitated transport of hydrophobic substrates across the outer cell membrane is important in whole‐cell biocatalysis. The co‐expression of the gene *alkL* encoding for an outer‐membrane transporter increased the uptake of hydrophobic substrates, thereby increasing the overall activity.^[^ ^38^, ^43^ ^]^ While AlkL is crucial for the efficient transport, particularly of larger substrates (>C_10_), we expected it might also facilitate the cellular uptake of the shorter but sterically hindered **1a**. Therefore, we expanded the *alkB(homolog)FGT* operon by the *alkL* gene. Figure 2a shows a clear trend of increased whole‐cell activity in the presence of AlkL for **1a** (up to 2‐fold) and *n*‐octane (up to 4‐fold; Figure S8A).

The engineering of membrane enzymes remains a challenge. Limited knowledge of their structure and reaction mechanisms poses an obstacle to their rational engineering.^[^ ^36^, ^37^, ^47^ ^]^ In lieu of accurate structural information on AlkB, random mutagenesis has been the method of choice so far. Two directed evolution studies based on error‐prone polymerase chain reaction (epPCR) of PpGPo1AlkB selecting variants with improved alkane utilization revealed that for one, substituting the bulky W55 in the substrate channel with a smaller serine shifts the substrate preference to longer alkanes ≥ C_12_. The triple‐variant V129M + L132V + I233V (MVV) showed higher activities toward short alkanes (C_4_–C_5_).^[^ ^47^, ^48^ ^]^ These variants presented themselves as starting points for the optimization of the biocatalyst. Recently, two cryo‐EM structures of AlkB from *Fontimonas thermophila* FtAlkB were published (PDB code: 8SBB and 8F6T), sharing about 60% sequence identity with PpGPo1AlkB. This allowed the selection of amino acid (aa) residues based on structural considerations.^[^ ^39^, ^40^ ^]^ The PpGPo1AlkB single variants W55S, V129M, L132V, I233V, as well as the triple variant MVV and MVV + W55S (SMVV), were tested in whole‐cell biotransformations (Figure 2b; Figure S8B).^[^ ^47^, ^48^ ^]^ In addition, a docking study was conducted based on the predicted AlphaFold2 (AF2) structure of PpGPo1AlkB.^[^ ^49^ ^]^ The two Fe‐atoms in the catalytic center, essential for the enzymatic activity and coordinated by four highly conserved His‐motifs, were modeled into the AF2‐structure (Figure 3a).^[^ ^37^, ^39^, ^40^, ^47^, ^54^ ^]^ **1a** was then docked into the inner catalytic pocket of PpGPo1AlkB. Residues lining the binding pocket, exhibiting high energies and closest to the best docking pose, were subjected to computational mutagenesis analysis using Rosetta FastDesign,^[^ ^55^ ^]^ whereby the sampled aa identities were restricted to hydrophobic residues only. The selected residues (N135, F164, L265, A268, and L309) in the active site are shown in Figure 3b. At position L309, no other energetically more favorable aa than Leu was identified during the Rosetta redesign. The two most frequently found single mutations for N135, F164, L265, and A268 were experimentally characterized. Investigation of the PpGpo1AlkB variants (Figure 2b) in whole‐cell biotransformations with **1a** and *n‐*octane (Figure S8B) revealed that the mutation I233V seems to be an activity driver for isoprenyl acetate and *n‐*octane. Interestingly, the mutation F164L resulted in higher activities toward **1a** but decreased the activity toward *n*‐octane.

**Figure 3:**
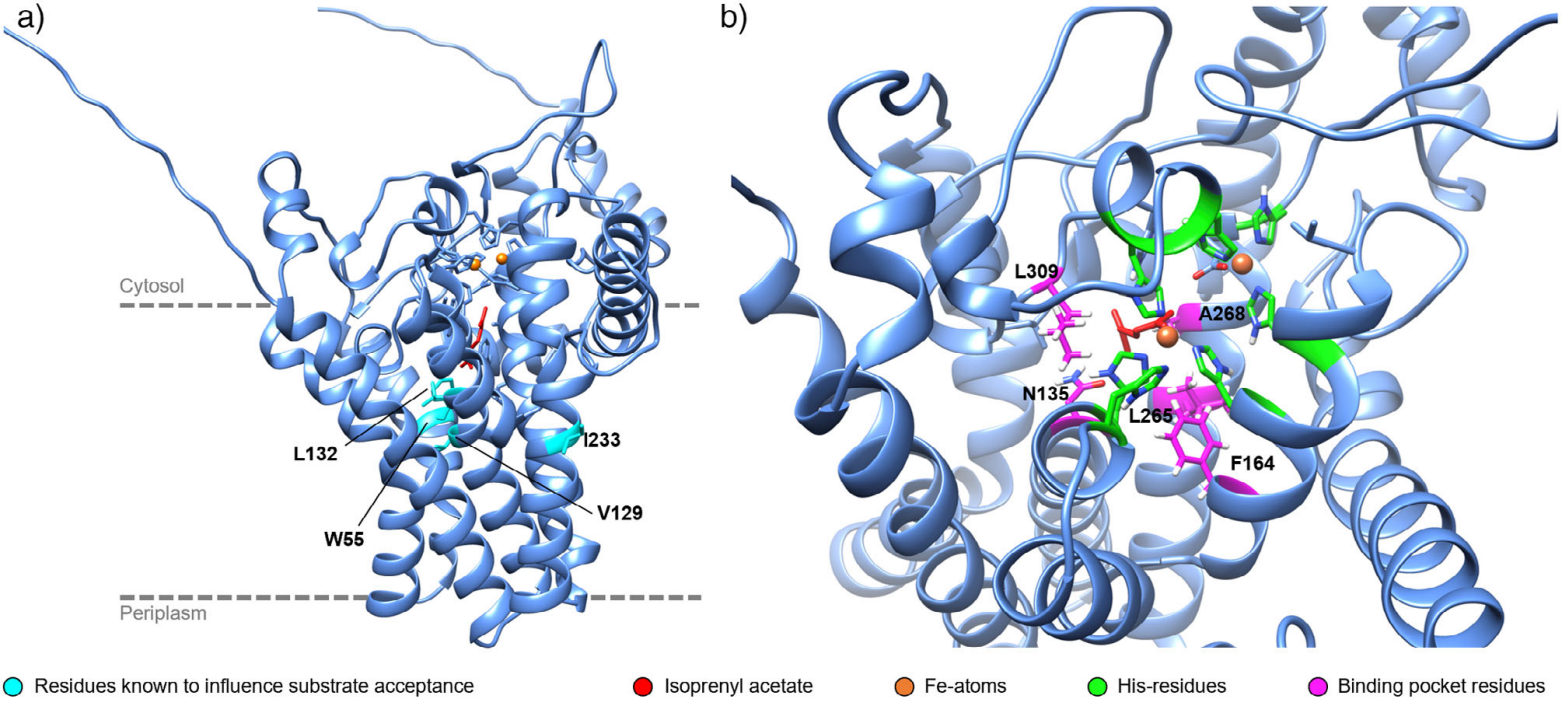
a) Design of PpGPo1AlkB based on the AF2‐model with the iron atoms (orange spheres) coordinated by highly conserved histidines in the catalytic center used for docking studies with isoprenyl acetate **1a** (red) and the prediction of active site mutations. The residues reported to influence substrate acceptance in PpGPo1AlkB (W55, V129, L132, and I233) are highlighted in cyan. The dashed grey line approximates the position of the enzyme in the inner cell membrane. b) Close‐up of the binding pocket of PpGPo1AlkB. Residues close to the docked substrate and showing high Rosetta Energy Units were selected as targets for mutation studies (N135, F164, L265, A268, and L309) and are shown in pink.

We further investigated the potential enhancement of **1b** formation by combining the engineering approaches in PpGPo1AlkB and M_AlkB. Surprisingly, no additive effect of the double mutation I233V + F164L was observed (Table 1; activity based on **1b** formation in the presence of AlkL). As expected, the co‐expression of *alkL* resulted in higher activities with I233V, F164L, and the combined variant. We were curious if the homologous substitutions I238V and F169L in M_AlkB in combination with AlkL would lead to a similar boost as for PpGPo1AlkB (Table 1). Indeed, the I238V and F169L variants were more active toward **1a** than the wildtype (WT), but no additive effect of the combined mutations (F169L + I238V) in M_AlkB was observed.

**Table 1:** Specific activities and relative activities (variant over WT) of PpGPo1AlkB and M_AlkB WT and variants (with AlkL present) toward **1a**. The substrate preference *P* represents the activity ratio with **1a** over *n‐*octane, while the preference shift compares the change in preference of the variant over the WT enzyme. The specific activity (µmol product formed per min and g_cdw_) is represented as arithmetic mean ± standard deviation (*n* = 3).

		Isoprenyl acetate 1a			
Enzyme	Variant (V)	Specific activity (U g_cdw_^−1^)	Relative activity	Substrate preference ***P*** ***v***_1*a*_/***v***_***n***−*octane*_	Preference shift ***P***_***V***_/***P***_***WT***_
**PpGPo1AlkB**	WT	0.51 ± 0.12	1	0.1	–
	I233V^[^ ^48^ ^]^	0.81 ± 0.28	1.61	0.1	2.1
	F164L	1.08 ± 0.13	2.1	0.2	3.1
	I233V + F164L	0.87 ± 0.19	1.71	0.1	2.0
**M_AlkB**	WT	1.02 ± 0.22	1	0.1	–
	I238V	1.83 ± 0.32	1.79	0.2	2.1
	F169L	0.91 ± 0.03	0.89	0.2	1.6
	I238V + F169L	0.63 ± 0.01	0.62	0.1	1.1

In nature, alcohol and aldehyde dehydrogenases (ADH and AlDH) are often combined for the oxidation of alcohols to carboxylic acids.^[^ ^56^ ^]^ In AlkB reactions with *n*‐octane, 1‐octanol was partially oxidized to the aldehyde and carboxylic acid by *E. coli* enzymes (Figure S8). In contrast, no oxidation of **1b** was observed either by native host enzymes or when the ADH and AlDH from the *alk‐*operon a*lkJ* and a*lkH* were co‐expressed. (Figure S6). This indicates that these enzymes do not act on **1b**, potentially due to the steric hindrance represented by the *exo*‐olefin group.

To identify suitable enzymes for the oxidation of **1b**, a panel of 20 recombinantly produced ADHs was screened in vivo for activity on the structurally similar **2b**, **3b,** and 1‐octanol as a reference substrate (Scheme 2, Figure S10). Five of the 20 ADHs that showed conversions with the tested substrates were subjected to protein purification and in vitro biocatalysis. Two enzymes, namely the well‐studied horse liver ADH (HLADH)^[^ ^57^ ^]^ and the geraniol dehydrogenase from *Castellaniella defragrans* (*Cd*GeDH),^[^ ^56^ ^]^ were active toward **2b** and **3b** in their purified form (Figures S12,S13). Using HLADH in combination with the water‐forming 1,4‐dihydro‐nicotinamide adenine dinucleotide (NADH) oxidase from *Lactobacillus pentosus* (*Lp*NOx)^[^ ^58^ ^]^ for NAD^+^ co‐factor recycling ∼50% and ∼92% conversions were achieved with **2b** and **3b** as substrates, respectively. *Cd*GeDH, combined with *Lp*NOx reached ∼84% with **2b**, but only ∼36% with **3b**. Interestingly, HLADH showed a tendency to further oxidize **3b** to **3d** (Figure S12). In the case of *Cd*GeDH, overoxidation of **2b** or **3b** was not observed.

**Scheme 2:**
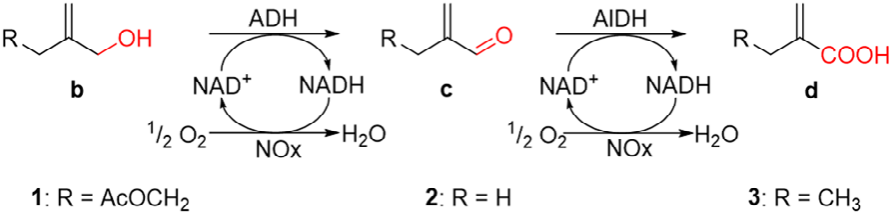
Oxidation of allylic alcohols **1b**, **2b**, and **3b** to the carboxylic acids by coupled alcohol and aldehyde dehydrogenase (ADH and AlDH), combined with co‐factor regeneration by water‐forming NADH oxidase from *Lactobacillus pentosus* (*Lp*NOx).

HLADH and *Cd*GeDH were then applied in the cell‐free oxidation of the allylic alcohol **1b**. We were pleased to find that both enzymes converted **1b** to the aldehyde **1c**, albeit without forming **1d** (Figure S14,S15). Hence, a second enzyme for the oxidation of the aldehyde was required. *Cd*GeDH was identified as part of an anaerobic catabolic pathway in *C. defragrans* of monoterpenoids by Harder et al. It oxidizes monoterpenols to monoterpenals, which are further converted to the carboxylic acids by the geranial dehydrogenase (*Cd*GaDH).^[^ ^56^ ^]^ When we applied *Cd*GaDH together with *Cd*GeDH or HLADH, **1b** was indeed converted to **1d** via **1c** (Figure S14,S15). In a cascade with *Cd*GeDH, *Cd*GaDH, and *Lp*NOx we reached ∼95% conversion of **1b** to **1d**. After acidification and incubation at 80 °C, an overall conversion of ∼83% (*n* = 2) of **1b** to **1e** was achieved (Figure 4).

**Figure 4:**
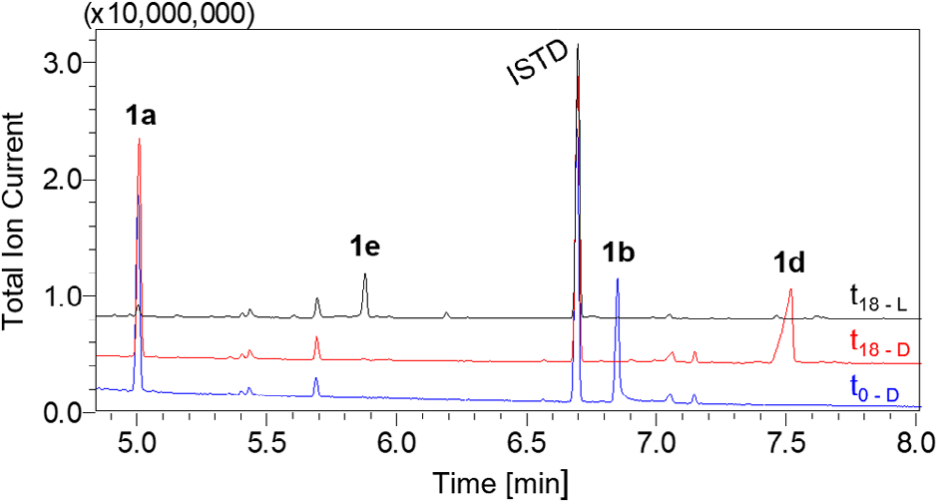
Oxidation of **1b** to **1d** by cell‐free enzyme cascade of *Cd*GeDH, *Cd*GaDH, and *Lp*NOx for NAD^+^ recycling. Directly extracted (D) reaction samples after 18h showed that **1b** was converted to **1d**. Lactonization (L) via acid catalysis gives rise to **1e**.

The knowledge gap in the natural biosynthesis of tulipalin A in plants has so far hindered its biotechnological production. In this work, we demonstrate for the first time the feasibility of its production from terpenoid precursors. The key is the combination of two challenging enzymatic reactions. First, the selective hydroxylation of **1a** in the terminal position without internal hydroxylation and epoxide formation, followed by the oxidation of the sterically demanding primary alcohol **1b** to **1d**. Bacterial alkane monooxygenases showed striking selectivity in the terminal hydroxylation of isoprenyl acetate without undesired side reactions. A combination of rational design, enzyme screening, and transporter co‐expression achieved a 6‐fold improvement for the selective hydroxylation. Even though only a few of the enzyme variants based on the de novo predicted structure showed the desired activity improvement. Due to the lack of a high‐throughput assay for non‐natural substrates, site‐directed mutagenesis of AlkB proved to be a practicable strategy. The activity improvement in relation to the WT enzymes is comparable to that achieved in the directed evolution of the activity toward *n*‐alkanes.^[^ ^47^, ^48^ ^]^ The identification of dehydrogenases acting on β‐substituted substrates was crucial for the oxidation of **1b** to **1d**. Assembled into a one‐pot two‐step cascade reaction, these enzymes represent a new pathway for the production of bio‐based tulipalin A. As the natural biosynthesis of tulipalin A remains elusive, our results provide a solid foundation for future research on the synthesis of this highly promising α‐methylene‐lactone.

## Supporting Information

The authors have cited additional references within the Supporting Information.^[^ ^59^, ^60^, ^61^, ^62^, ^63^, ^64^, ^65^, ^66^, ^67^, ^68^, ^69^, ^70^, ^71^, ^72^, ^73^, ^74^, ^75^, ^76^, ^77^, ^78^, ^79^, ^80^, ^81^, ^82^, ^83^, ^84^, ^85^ ^]^

## Conflict of Interests

The authors declare no conflict of interest.

## Supporting information



Supporting information

## Data Availability

The data that support the findings of this study are available from the corresponding author upon reasonable request.
