# Supplementary material for: Engineering of Transmembrane Alkane Monooxygenases to Improve a Key Reaction Step in the Synthesis of Polymer Precursor Tulipalin A
Source: Angew Chem Int Ed Engl. 2025 May 16;64(25):e202503464. doi: 10.1002/anie.202503464 (PMC12171333; doi:10.1002/anie.202503464)
Supplement: Supplementary file 1 — Supporting information [file ANIE-64-e202503464-s001.pdf]

Supporting Information

Engineering of Transmembrane Alkane Monooxygenases to Improve a Key Reaction Step in the Synthesis of Polymer Precursor Tulipalin A

Andrea Nigl,[a,b] Veronica Delsoglio,[c] Lucija Sovic,[a] Marina Grgić,[a] Lenny Malihan-Yap,[a] Kamela Myrtollari,[a,e] Jelena Spasic,[a] Margit Winkler,[a,b] Gustav Oberdorfer,[c,d] Andreas Taden,[e] Iva Anić,[e] Robert Kourist[a,b,d*]

[a] Graz University of Technology, Institute of Molecular Biotechnology, Petersgasse 14, 8010 Graz, Austria

[b] acib GmbH (Austrian Centre of Industrial Biotechnology), Petersgasse 14, 8010 Graz, Austria

[c] Graz University of Technology, Institute of Biochemistry, Petersgasse 12/2, 8010 Graz, Austria

[d] BioTechMed-Graz, Mozartgasse 12/II, 8010 Graz, Austria.

[e] Henkel AG & Co. KGaA, Henkel-Str. 67, 40589 Düsseldorf, Germany

[*] E-Mail: kourist@tugraz.at

Content

1 Computational methods 2
2 Chemicals and devices 3
2.1 Synthesis of isoprenyl acetate 1a 3
2.2 Synthesis of 4-acetoxy-2-methylene butyric acid 1d 3
2.3 Synthesis of 3-methyl-3,4-epoxy butyl acetate 1f 3
3 Genes and plasmids 3
3.1 Cloning *alk*-operon 3
3.2 Sequences ADHs and AlDHs 4
4 Media preparation 5
5 Biocatalytic reactions employing unspecific peroxygenases 6
6 Expression of *alk*-operon and whole-cell biotransformation 6
7 ADH and AlDH screening, production, and biotransformation 7
7.1 ADH deep well plate screening 7
7.2 ADH production and conversion of 2b and 3b 7
7.3 *Cd*GaDH production 8
7.4 *In vitro* oxidation of 1b by ADH-AlDH cascade 8
8 Analytics 9
8.1 Lactonization of 1d to 1e 9
8.2 Analysis by gas chromatography (GC) 9
8.3 High-performance liquid chromatography (HPLC) analysis 10
8.4 NMR-spectroscopy 11
9 Synthesis of 1f 11
10 UPO-catalyzed transformation of isoprenyl acetate 11
11 alkBFGT*(HJL)* expression and whole-cell biotransformations 12
11.1 Control reaction with isoprenol 5a 15
12 ADH screening 16
13 Shake flask production of ADHs and AlDHs 16
14 2b and 3b conversion by HLADH and *Cd*GeDH 17
15 1b oxidation by ADH-AlDH cascade and lactonization to 1e 19

1 Computational methods

The workflow of the docking and computational mutagenesis study is shown in Figure S1. The docking study began by preparing the protein scaffolds and ligands (isoprenyl acetate, and *n*-octane). Protein scaffolds for PpGPo1AlkB and M_AlkB were constructed using AlphaFold v2.3.1 (AF2), ensuring 50%-70% sequence coverage with pLDDT scores >90%. Using Rosetta, the iron atoms were added to the protein scaffold by placing two additional virtual atoms to the iron ligand params files to ensure constraint geometries were measured accurately.[59] This parameter file was used for matching to the AF2 predicted structure. Metal atom placement was finalized using RosettaScripts employing the AddOrRemoveMatchCsts and EnzRepackMinimize movers to ensure correct coordination to the histidine residues – 138 and 273 for PpGPo1AlkB and 143 and 278 for M_AlkB.

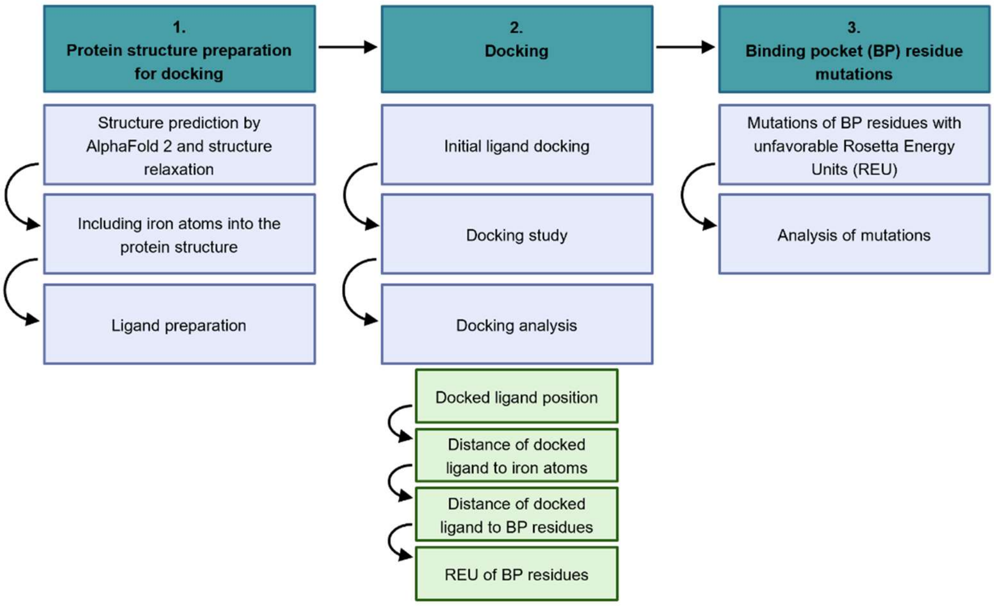

Figure S1. Workflow of docking and computational mutagenesis in Rosetta based on the AlphaFold 2 predicted structures of PpGPo1AlkB and M_AlkB.

Parameter and coordinate files of the ligand molecules were downloaded from PubChem in XML and PDB formats. The files were converted to mol2 format using OpenBabel,[60] followed by conversion to Rosetta params files using the molefile_to_params.py script, as provided with the Rosetta modeling suite. Initial ligand placement was performed via geometry-based molecular docking using the PatchDock server, with the best-scored ligand pose selected for further high-resolution docking. High-resolution docking was restricted to the central pocket, near the iron atoms, due to constraints such as the protein models' rigidity and lack of favorable docking regions elsewhere, aiming to identify important residues for enhancing catalytic activity. RosettaScripts was used to assemble a full docking protocol, which included initial minimization and repacking of the protein scaffold, followed by the execution of the GALigandDock[61] mover for docking, and concluded with a final round of structure minimization and repacking. Data analysis filtered out ligands docked outside the protein scaffold. Evaluation of docked ligands relied on the Rosetta Ligand score and geometries of the docked structure. The total score of the best-scoring molecules was used for further analysis. Approximately 90% of the docked ligands retained final positions within the protein scaffold. This enabled the identification of potential key residues for enzymatic activity by the docking of isoprenyl acetate and *n*-octane, respectively. These residues were identified via their distances between the terminal atoms in the docked ligand poses and the surrounding residues. For PpGPo1AlkB, surrounding residues included TRP 55, ILE 128, LEU 132, ASN 135, PHE 164, GLN 264, LEU 265, ALA 268, ASN 269, ILE 271, VAL 305, LEU 306, LEU 309, TYR 339, and PHE 343. For M_AlkB, they comprised GLY 136, LEU 139, PHE 166, TYR 170, GLU 172, GLU 203, ILE 204, ALA 207, PHE 208, TRP 267, LEU 270, THR 271, and ASN 274. The nearest residues to the ligands' terminal carbon atoms were identified to be ASN 135, PHE 164, LEU 265, ALA 268, and LEU 309 for PpGPo1AlkB and GLY 136, LEU 139, TYR 169, ILE 204, LEU 270, and ASN 274 for M_AlkB. Consistency was ensured by calculating the average distances between these residues and ligand atoms of the best-scored ligands. Additionally, we assessed the Rosetta score per residue of these closest residues. For PpGPo1AlkB, ASN 135, LEU 265, LEU 309, and TYR 339 exhibited positive scores, indicating unfavorable energetic conditions for ligand docking. For M_AlkB, GLY 136 and ILE 204 showed similar unfavorable conditions, guiding the selection process for key residues involved in catalysis.

The selected residues were optimized via RosettaScripts utilizing the FastDesign mover, which selectively redesigns them using a limited amino acid alphabet. Output sequences are compared to evaluate the frequency of occurrence for each substituent, indicating structural favorability. For PpGPo1AlkB, the analysis reveals LEU (91%) and PHE (3%) as the most frequent residue at positions 135, LEU (65%) and TRP (8%) at 164, TRP (54%) and TYR (7%) at position 265, and ILE (79%) and PHE (10%) at position 268, while the native amino acid (LEU) remains predominant at position 309.

The full code is available under: https://github.com/veronicadelsoglio-code/Docking-study-AlkB/blob/main/Mutation.xml.

2 Chemicals and devices

If not stated otherwise, all chemicals and compounds used in this work were either purchased from Merck/Sigma-Aldrich, TCI chemicals, or Carl Roth in the highest purity available. Standard laboratory equipment wasused for this study.

2.1 Synthesis of isoprenyl acetate 1a

1a was synthesized from isoprenol **5a** similarly as described by Bartoli and co-workers.[62] To a 50 mL round-bottom flask equipped with magnetic stirring, 60.9 mmol of Ac_2O and 0.58 mmol of $Mg(ClO)_4$ were added. The reaction was initiated by a dropwise addition of **5a** (58 mmol). The reaction was monitored by thin layer chromatography (TLC; cyclohexane/EtOAc 3:1) until completion (approx. 30 min). Aqueous $NaHCO_3$ was added, and the product was extracted with Et_2O. The organic layer was dried over $MgSO_4$ and the solvent was evaporated to give the pure acetate ester (yield: 88%). The formation of **1a** was verified by gas chromatography-mass spectrometry (GC-MS) and nuclear magnetic resonance (NMR) spectroscopy and compared to reported spectroscopic data (^{1}H NMR (300 MHz, $CDCl_3$), *δ* 4.81 (s, 1H), 4.74 (s, 1H), 4.18 (t, *J* = 6.87 Hz, 2H), 2.34 (t, *J* = 6.72 Hz, 2H), 2.04 (s, 3H), 1.76 (s, 3H) ppm).[63]

2.2 Synthesis of 4-acetoxy-2-methylene butyric acid 1d

In a 10 mL round bottom flask equipped with a magnetic stirrer, 200 mg (1.7 mmol) of 2-methylene-4-hydroxy butyric acid **5d** (Enamine, Kyiv, Ukraine) was selectively acetylated to 4-acetoxy-2-methylene butyric acid **1d** with acetic anhydride (5.0 eq.) and pyridine (5.7 eq.) at 0 °C overnight. After completion of the reaction (monitored by TLC), the reagents were initially evaporated *in vacuo*. The obtained residue was washed with 1 M aq. HCl (5 mL) and extracted with EtOAc (3 x 5 mL). The combined organic layers were concentrated under reduced pressure in a water bath at 50 °C. Further evaporation under inert gas conditions to remove remaining traces of reagents yielded 4-acetoxy-2-methylene butyric acid **1d** in pure form as a yellow, viscous oil (167 mg, 62% yield). NMR data of the product **1d** is in total agreement with the literature (^{1}H NMR (300 MHz, $CDCl_3$), *δ* 6.37 (s, 1H), 5.73 (s, 1H), 4.23 (t, *J* = 6.6 Hz, 2H), 2.65 (t, *J* = 6.6 Hz, 2H), 2.03 (s, 3H) ppm).[64]

2.3 Synthesis of 3-methyl-3,4-epoxy butyl acetate 1f

2-(2-Methyl-2-oxiranyl)ethanol **5f** (Enamine, Kyiv, Ukraine) was acetylated using a lipase as the catalyst to the product 3-methyl-3,4-epoxy butyl acetate **1f** using vinyl acetate as acetate donor. Briefly, 20.4 mg (0.20 mmol) of **5f** was reacted with 37 µL (0.40 mmol) of vinyl acetate in dry methyl tert-butyl ether (MTBE) following previously published methods.[46,65] 1.62 mg lipase from *Pseudomonas cepacia* (>30 U mg^{-1}, Sigma-Aldrich) was added, and the reaction mixture was performed at 30 °C and 500 rpm for 24 h. The reaction mixture was centrifuged, and the supernatant was concentrated using a rotary evaporator. Analysis of the reaction mixture was performed using GC-MS and the formation of **1f** was confirmed by NMR spectroscopy. (^{1}H NMR $CDCl_3$, 300 MHz: *δ* 1.29, (s, 3H), *δ* 1.74-1.94 (m, 2H), *δ* 1.99 (s, 3H), *δ* 2.53 (d, 1H, *J* = 6 Hz), *δ* 2.58 (d, 1H, *J* = 4.6), *δ* 4.00-4.19 (m, 2H) ppm).

3 Genes and plasmids

3.1 Cloning *alk*-operon

Synthetic DNA-fragments encoding *alkBFG*, *alkT, alkJ,* and *alkH* from *P. putida* GPo1 were ordered from Integrated DNA Technologies (IDT; Leuven, Belgium). The sequence information was taken from the *P. putida* OCT plasmid *alk* genes cluster (NCBI nucleotide: AJ245436.1). The fragments were sub-cloned into the pJET1.2-vector using the Thermo Scientific CloneJET PCR Cloning Kit. The *PpGpo1alkBFG* and *PpGpo1alkT* fragments were inserted into the pCom10 vector backbone[45] via the FastCloning method.[66] The operon of the p*PpGpo1alkB-FGT* vector was expanded with *PpGpo1alkL, PpGpo1alkHJ, PpGpo1alkHJL,* and *PpGpo1alkJ,* resulting in p*PpGpo1alkB-FGTL,* p*PpGpo1alkB-FGTHJ,* p*PpGpo1alkB-FGTHJL, and* p*PpGpo1alkB-FGTJ,* respectively. The *alkL* gene was amplified from the pCom10_*alkL*[45] adding a ribosome binding site (RBS) upstream of *PpGpo1alkL* and subsequently cloned into the p*PpGpo1alkBFGT* via FastCloning. The constructs p*PpGpo1alkBFGTHJ,* p*PpGpo1alkBFGTHJL, and* p*PpGpo1alkBFGTJ* were generated via Gibson Assembly by expanding the operon with the respective fragments as reported by Nuland *et al*.[38,67]

Four additional genes encoding homologous AlkB alkane monooxygenases (MO) from other organisms were also ordered as synthetic DNA-fragments from IDT: a) AlkB from *Pseudomonas putida* P1 (PpP1AlkB; NCBI protein: CAB51047.1),[50] b) AlkB1 from *Alcanivorax borkumensis* (AboAlkB; NCBI protein: BAC98365.1),[51] c) AlkB from *Acinetobacter baylyi* (AbaAlkB; NCBI protein: WP_120429654.1) and d) M_AlkB from *Marinobacter* sp. (M_AlkB; NCBI protein: MAB50652.1). The genes of the homologous alkane MOs were cloned into the p*PpGpo1alkBFGT* vector via Gibson Assembly replacing the *PpGpo1alkB* while leaving the electron transfer system *PpGPo1alkFGT* intact*.* The constructs encoding the homologs were also expanded by the *alkL* gene for co-expression of the transporter. Correct assembly was verified by Sanger Sequencing (Microsynth, Austria). Table S1 lists all plasmids used in this study to clone and recombinantly express the *alk*-operon in *E. coli* BL21(DE3). Single and combinatorial mutants of *PpGPo1alkB* and *M_alkB* (Table S2) were generated by site-directed mutagenesis (SDM). The non-active variant H273A was generated for control experiments. Successful mutagenesis was verified by Sanger sequencing.

Table S1. Listing of plasmids used for cloning and expressing the *alk*-operon. All plasmids for the expression of *alkB(homolog/mutant)* are based on the broad-host vector pCom10 employing an alkane-inducible promotor system.[45] The inserts have been ordered as synthetic genes and cloned into the pCom10-backbone either by the FastCloning method or Gibson Assembly.

**Name**	**Gene origin**	**Backbone**	**Purpose**
p*PpGpo1alkB-FGT*	*P. putida* GPo1	pCom10	Expression of *PpGpo1alkBFGT*
p*PpGpo1alkB-FGTL*	*P. putida* GPo1	pCom10	Expression of *PpGpo1alkBFGTL*
p*PpGpo1alkB-FGTHJ*	*P. putida* GPo1	pCom10	Expression of *PpGpo1alkBFGTHJ*
p*PGpo1alkBF-GTHJL*	*P. putida* GPo1	pCom10	Expression of *PpGpo1alkBFGTHJL*
p*PpGpo1alkB-FGTJ*	*P. putida* GPo1	pCom10	Expression of *PpGpo1alkBFGTJ*
p*PpP1alkB-FGT*	*P. putida* P1	p*PpGpo1alkB-FGT*	Expression of *PpP1alkB-PpGpo1alkFGT*
p*M_alkB-FGT*	*Marinobacter sp.*	p*PpGpo1alkB-FGT*	Expression of *M_alkB-PpGpo1alkFGT*
p*AboalkB-FGT*	*A. borkumensis*	p*PpGpo1alkB-FGT*	Expression of *AboalkB-PpGpo1alkFGT*
p*AbaalkB-FGT*	*A. baylyi*	p*PpGpo1alkB-FGT*	Expression of *AbaalkM-PpGpo1alkFGT*
p*PpP1alkB-FGTL*	*P. putida* P1	p*PpGpo1alkB-FGTL*	Expression of *PpP1alkB-PpGpo1alkFGTL*
p*M_alkB-FGTL*	*Marinobacter sp.*	p*PpGpo1alkB-FGTL*	Expression of *M_alkB-PpGpo1alkFGTL*
p*AboalkB-FGTL*	*A. borkumensis*	p*PpGpo1alkB-FGTL*	Expression of *AboalkB-PpGpo1alkFGTL*
p*AbaalkB-FGTL*	*A. baylyi*	p*PpGpo1alkB-FGTL*	Expression of *AbaalkB-PpGpo1alkFGTL*
pCom10_*alkL*	*P. putida* GPo1	pCom10	Backbone for cloning and negative control (EVC)
pJET1.2_*alkBFG*	*P. putida* GPo1	pJET1.2	Cloning of synthetic DNA fragments
pJET1.2_*alkT*	*P. putida* GPo1	pJET1.2	Cloning of synthetic DNA fragments
pJET1.2_*alkH*	*P. putida* GPo1	pJET1.2	Cloning of synthetic DNA fragments
pJET1.2_*alkJ*	*P. putida* GPo1	pJET1.2	Cloning of synthetic DNA fragments

Table S2. Listing of all mutants of *PpGpo1AlkB* and *M_AlkB* tested in this work. Mutants were created by SDM using p*PpGpo1alkB-FGT* or p*M_alkB-FGT* as a template. Selected mutations (*) were also introduced in p*PpGpo1alkB-FGTL* and p*M_alkB-FGTL*.

**Variants PpGpo1AlkB**	**Ref.**	**Variants PpGpo1AlkB**	**Ref.**
W55S	[47]	L265W	this study
V129M	[48]	L265Y	this study
L132V	[48]	A268I	this study
I233V*	[48]	A268F	this study
H273A	[47]	F164L + I233V (LV)*	this study
V129M + L132V + I233V (MVV)	[48]	**Variants M_AlkB**	
W55S + V129M + L132V + I233V (MVV)	[47,48]	F169L*	this study
N135L	this study	I238V*	this study
N135F	this study	F169L + I238V (LV)*	this study
F164L*	this study		
F164W	this study		

3.2 Sequences ADHs and AlDHs

Table S3 provides an overview of the alcohol dehydrogenase (ADHs) and aldehyde dehydrogenase (AlDHs) constructs tested in this work. The plasmid encoding ADH #1 was thankfully provided by Dr. A. Pick (Cascat GmbH, Straubing, Germany). The constructs ADH #2-4 are derived from unpublished work. ADH #7-20 were selected based on extensive literature and database searches based on substrate scope and co-factor preferences. The genes for ADH #7-20 were codon-optimized for *E. coli* and ordered pre-cloned into the multiple cloning site I of the pCDFDuet vector with an N-terminal 6xHis-tag from BioCat GmbH (Heidelberg, Germany). The gene encoding the AlDH *Cd*GaDH was codon-optimized for *E. coli* expression and ordered from Twist Bioscience (San Francisco, United

States) pre-cloned in-frame with the N-terminal His-tag. pCDFDuet and pET28a(+) without inserts were used as empty vector controls (EVC).

Table S3. ADH and AlDH constructs used in this study. Empty pCDFDuet and pET28a(+) were used for negative controls.

ADH #	Abbrev.	Enzyme	Origin	NCBI accession/ SeqID	Vector backbone	Ref.
1	AdhZ3-LND	Aldehyde reductase Ahr	*Escherichia coli*	AFG43188.1	pET28a(+)	[68]
2	AdhA	Aldehyde reductase AdhA	*Synechocystis sp.PCC 6803*	WP_010874320.1	pET28a(+)	[69]
3	AdhB	Putative aklaviketone reductase	*Synechocystis sp.PCC 6803*	WP_041425929.1	pET28a(+)	–
4	AdhC	Short-chain alcohol dehydrogenase	*Synechocystis sp.PCC 6803*	WP_010872685.1	pET28a(+)	–
5	SDR-B6	Short chain dehydrogenase	*Cupriavidus necator*	WP_011616349.1	pK470	[70]
6	HisSDR-B6	Short chain dehydrogenase (6xHis-N-term)	*Cupriavidus necator*	WP_011616349.1	pK470	[70]
7	CdGeDH	Geraniol dehydrogenase	*Castellaniella defragrans*	CCF55024.1	pCDFDuet	[56]
8	ChnD	Alcohol dehydrogenase	*Acinetobacter sp.* NCIMB 98713	BAC80217.1	pCDFDuet	[71]
9	TgGhbd1	γ-hydroxybutyrate dehydrogenase	*Tulipa gesneriana*	SEQ-ID No 13*	pCDFDuet	[18]
10	AtGlyR1	Glyoxylate/succinic semialdehyde reductase 1	*Arabidopsis thaliana*	NP_566768.1	pCDFDuet	[72]
11	AcAlrA	NADP-dependent alcohol dehydrogenase	*Acinetobacter sp. M-1*	BAB12270.1	pCDFDuet	[73]
12	HLADH	Alcohol dehydrogenase E chain	*Equus caballus*	AAA30931.1	pCDFDuet	[57]
12	HLADH	Alcohol dehydrogenase E chain	*Equus caballus*	AAA30931.1	pET28a(+)	[57]
13	CmCAD1	Putative cinnamyl alcohol dehydrogenase 1	*Cinnamomum micranthum*	RWR78051.1	pCDFDuet	[74]
14	EcYqhD	Alcohol dehydrogenase YqhD	*Escherichia coli*	AAA69178.1	pCDFDuet	[75]
15	YsADH	Alcohol dehydrogenase	*Yokenella sp. WZY002*	AHI87872.1	pCDFDuet	[76]
16	ZoGeDH	cinnamyl-alcohol dehydrogenase	*Zingiber officinale*	BAR42579.1	pCDFDuet	[77]
17	PpBDH	Aryl-alcohol dehydrogenase	*Pseudomonas putida*	BAA09664.1	pCDFDuet	[78]
18	RoGDH	Geraniol dehydrogenase	*Rhodococcus opacus*	AII08455.1	pCDFDuet	-
19	TcADH1	Alcohol dehydrogenase 1	*Tanacetum cinerariifolium*	AUQ44117.1	pCDFDuet	[79]
20	ClGDH	Geraniol dehydrogenase	*Carpoglyphus lactis*	BAG32342.1	pCDFDuet	[80]
**AlDH #**	**Abbrev.**	**Enzyme**	**Origin**	**NCBI accession/ SeqID**	**Vector backbone**	**Ref.**
1	CdGaDH	Geranial dehydrogenase	*Castellaniella defragrans*	CCF55023.1	pET28a(+)	[56]

*sequence derived from WO2002101013A2[18]

4 Media preparation

For the expression of the AlkB-system and the screening of the ADHs, an adapted M9 minimal media (M9MM) or an M9-based autoinduction media (M9AIM) were used.[81] The components of the media were prepared and sterilized separately as described in Table S4. For 1 L of M9MM 200 mL of 5x M9-salts, 2 mL of 1 M $MgSO_4$, 1 mL of thiamine, 50 µL of biotin, 1 mL of US^{Fe} trace element solution, and 25 mL of glucose solution were mixed and filled up to 1 L with autoclaved ddH_2O. For 1 L M9AIM 20 mL of 50x 5052 were added instead of glucose.

Table S4. Components of M9 minimal medium (M9MM) and M9-based autoinduction medium (M9AIM).

Component	Final conc.	Stock	Stock preparation
**Na_2HPO_4*2 H_2O**<br>**KH_2PO_4**<br>**NaCl**<br>**NH_4Cl**	8.5 g L^{-1}<br>3.0 g L^{-1}<br>0.5 g L^{-1}<br>2.0 g L^{-1}	5x M9-salts	pH adjusted to 7.0 with 5 M NaOH; autoclaved; stored at room temperature
**$MgSO_4$*7 H_2O**	2 mM	1 M	Autoclaved, stored at room temperature
**Thiamine*HCl**	1 mg L^{-1}	1000x	50 mg dissolved in 50 mL ddH_2O and filter sterilized; stored -20°C
**Biotin**	5 µg L^{-1}	20000x	5 mg dissolved in 45 mL ddH_2O, 1 N NaOH added until dissolved, filled up to 50 mL with ddH_2O and filter sterilized, stored at -20 °C
**US^{Fe} trace element solution***	1 mL L^{-1}	1000x	All components below dissolved in 1 M HCl; filter sterilized; stored at -20°C in 1 mL aliquots
**Glucose**	5.0 g L^{-1}	20%	Dissolved in 1 L ddH_2O; autoclaved; stored at 4 °C
**Glycerol**<br>**Glucose**<br>**α-lactose**	5.0 g L^{-1}<br>0.5 g L^{-1}<br>2.0 g L^{-1}	50x 5025	Add in sequence and dissolve stepwise in ddH_2O, fill up to 1 L and filter sterilize. Store at 4°C

***1000x US^{Fe} trace element solution**

Compound	Stock conc.	Compound	Stock conc.
$FeSO_4$*7 H_2O	8.87 g L^{-1}	H_3BO_3	0.30 g L-1
$CaCl_2$*2 H_2O	4.12 g L^{-1}	Na_2MoO_4*2 H_2O	0.25 g L^{-1}
$MnCl_2$*2 H_2O	1.23 g L^{-1}	$CuCl_2$*2 H_2O	0.15 g L^{-1}
$ZnSO_4$*7 H_2O	1.87 g L^{-1}	Disodium EDTA*2 H_2O	0.84 g L^{-1}

5 Biocatalytic reactions employing unspecific peroxygenases

The enzyme panel consisting of 77 unspecific peroxygenases (UPOs) was purchased from Aminoverse (Nuth, The Netherlands). The kit contains 62 wildtype and 15 mutant fungal UPOs (Table S5) produced as a secreted protein in *Pichia pastoris* and available in lyophilized form (1 mg). Isoprenyl acetate (100 mM) was prepared in acetonitrile and the co-substrate H_2O_2 (55 mM) in 1x tricine buffer (100 mM, pH 7.5). The reaction containing 195 µL buffer, 25 µL resuspended UPO solution, and 10 µL **1a** stock was initiated by adding 5 µL H_2O_2 stock. The reaction vial was placed in a thermoshaker operated at 30 °C and 500 rpm. 5 µL H_2O_2 was pulsed every 30 minutes in the first 2 h, reaching a final concentration of 4.4 mM. After 4 h, the reaction was quenched by adding the UPO-STOP solution (2 µL) containing catalase and incubated at room temperature for at least 15 min. The reaction mixture was then extracted with EtOAc containing 1 mM of methyl benzoate as an internal standard (ISTD) for GC-MS measurements.

A large-scale reaction (155x) was set up to confirm the structure of the UPO product by NMR-spectroscopy. Conversion of **1a** by UPO 12 Aminoverse/ UPO 13 Bisy (thankfully provided by Prof. Dr. Anton Glieder) was performed at 30 °C using 1.1 eq. of H_2O_2. The product was extracted and purified from the reaction mix. After 24 h, the reaction was quenched with the addition of catalase (43 µL, 20,000 U mL^{-1}). The product was then extracted using dichloromethane (1:2 v/v, 6x) and subjected to GC-MS and ^{1}H NMR analysis.

Table S5. Plate layout of UPO Enzyme panel (Aminoverse (Nuth, The Netherlands)).

UPO 1	UPO 5	UPO 3	UPO 21	UPO 29	UPO 42	**Aae UPO***	UPO 56	UPO 13M2	UPO 36M1
UPO 4	UPO 8	UPO 28	UPO 24	UPO 32	UPO 38	UPO 49	UPO 57	UPO 13M5	UPO 36M2
UPO 7	UPO 11	UPO 31	UPO 19	UPO 26	UPO 44	UPO 50	UPO 58	UPO 13M4	UPO 36M3
UPO 10	UPO 14	UPO 6	UPO 34	UPO 45	UPO 27	UPO 51	UPO 59	UPO 13M7	UPO 36M4
UPO 13	UPO 17	UPO 9	UPO 37	UPO 46	UPO 48	UPO 52	UPO 60	UPO 13M8	UPO 36M5
UPO 16	UPO 20	UPO 12	UPO 39	UPO 35	UPO 30	UPO 53	UPO 61	UPO 13M9	neg. c. **
UPO 22	UPO 25	UPO 15	UPO 41	UPO 47	UPO 33	UPO 54	UPO 13M3	UPO 13M10	UPO 13
UPO 2	UPO 23	UPO 18	UPO 43	UPO 40	UPO 36	UPO 55	UPO 13M1	UPO 12M11	-

*AaeUPO from *Agrocybe aegerite*; ** negative control without UPO; M: UPO-mutant

6 Expression of *alk*-operon and whole-cell biotransformation

E. coli BL21(DE3) harboring the respective plasmid (Table S1, Table S2) were used for alkB(homolog/mutant)-FGT*(HJL)* expression. The conditions were set similarly to those described in the literature.[38,42] *E. coli* BL21(DE3) pCom10_*alkL* was used for EVC experiments. Strains were grown in either LB or M9MM (Table S4) supplemented with 50 µg mL^{-1} kanamycin. 5 mL of LB medium was inoculated with a single colony and incubated overnight at 30 °C and constant agitation (120 rpm). 200 µL of the overnight culture

(ONC) were transferred to 20 mL of M9MM in a 100 mL baffled shake flask and the culture was incubated overnight at 30 °C. For the expression, 200 mL of M9MM were inoculated to an OD_{600} of 0.15 with the M9-preculture and grown at 30 °C until an OD_{600} of 0.4 to 0.5. By adding 0.05% (v/v) dicyclopropyl ketone (DCPK) recombinant gene expression was induced. After 4 h of expression at 30 °C the cells were harvested by centrifugation (4,400 x *g*, 4 °C, 15 min). The production of the recombinant proteins was verified by sodium dodecyl sulfate polyacrylamide gel electrophoresis (SDS-PAGE). After harvest, the cells were resuspended in resting cell buffer (RCB; 50 mM KPi, pH 7.4, 1% glucose, 2 mM $MgSO_4$) to an OD_{600} of 10 if not stated otherwise. The reaction mixture was prepared on ice as a homogenous master mix for each time-lapse sample. The reaction was initiated by the addition of 5 mM of substrate (isoprenyl acetate or *n*-octane; 200 mM stock in EtOH; 2.5% (v/v) EtOH in the reaction) and then split to 300 µL each into 1.5 mL tightly sealed glass vials, which were placed lying in a specialized rack and incubated at 25 °C under constant agitation (180 rpm). 250 µL were sampled (usually after 0, 15, 30, 60, 180 min, and 24 h) and quenched by adding 25 µL of 2 M HCl. The samples were stored at -20 °C until GC analysis. The biotransformations were performed in independent biological triplicates.

To identify the product formed by PpGPo1AlkB when using isoprenyl acetate **1a** as substrate, the reaction was scaled up to a total volume of 20 mL in an Erlenmeyer flask sealed with a metal cap and Parafilm®. The reaction was initiated by adding 5 mM of **1a** and performed at 25 °C and 180 rpm. The reaction was followed by GC-MS analysis. After 48 h, the product was extracted with EtOAc from the reaction mixture. The organic layer was dried over $MgSO_4$ and the solvent evaporated. **1b** was purified from the crude extract via column chromatography (cyclohexane/EtOAc 4:1) (Yield: < 5%; TLC: cyclohexane/EtOAc (2:1)). ^{1}H NMR was recorded in $CDCl_3$.

The supernatant of 20 mL reactions with resting *E. coli* BL21 (DE3) p*M_alkB(I238V)-FGTL* and *E. coli* BL21 (DE3) pCom_*alkL* (EVC) was used as substrates for the *in vitro* oxidation cascade (section 7.4). The reaction was set up as described in the paragraph above. After 18 h of reaction at 25 °C and 180 rpm, the supernatant was separated from the cells via centrifugation at 8,200 x *g*, 45 min, and 4 °C. The supernatant was either used directly for the oxidation reaction or stored at -20 °C for up to one week.

7 ADH and AlDH screening, production, and biotransformation

7.1 ADH deep well plate screening

E. coli K-12 MG1655 RARE[82] harboring the ADH plasmids listed in Table S3 were used for whole-cell screening in 96-deep well plates (DWPs). Therefore, 600 µL of LB medium supplemented with 40 µg mL^{-1} kanamycin (pET28a(+) and pK740) or 50 µg mL^{-1} streptomycin (pCDFDuet) per well were inoculated from the respective glycerol stock in triplicates. In addition, controls with empty pET28a(+) and pCDFDuet and sterile medium were prepared. The DWP was incubated overnight at 37 °C and 320 rpm at a 60 ° angle. The next day, a fresh DWP containing 600 µL M9AIM per well was inoculated using 10 µL of seed culture and supplemented with the corresponding antibiotic. The main cultures were grown for 4 h at 37 °C and 320 rpm and a 60 ° angle until they reached an OD_{600} of 0.5 to 0.6. Then the DWPs were transferred to a 20 °C shaker for the gene expression and agitated for 20 h at 320 rpm. Cells were harvested via centrifugation at 4,000 rpm and 4 °C for 0.5 h using Eppendorf 5810 Benchtop Centrifuge.

For the whole-cell biotransformation, the cell pellets were directly resuspended in 200 µL of 1X M9 salts supplemented with 1% glucose to ensure the co-factor regeneration. The whole-cell suspension was transferred to a 96-well microtiter plate (MTP) and the reaction was started by adding **2b** or **3b** to a final concentration of 2.5 mM from a stock (50 mM in DMSO). The MTP was agitated on a platform Heodolph Titramax 1000 at 340 rpm and room temperature. In addition to the target substrates, 1-octanol was used as a positive control. After 20 h of biocatalysis, reactions were stopped by adding 1/10 of 2 M HCl. To reaction samples with **2b** and **3b**, 250 µL methanol was added and prepared for HPLC analysis. Reaction samples with 1-octanol were extracted with 250 µL EtOAc with ISTD (1 mM 1-decanol) for GC-FID measurement.

7.2 ADH production and conversion of 2b and 3b

The alcohol dehydrogenases that showed conversion of 1-octanol, **2b**, and/or **3b** in the initial screening were subjected to shake flask expression and subsequent purification using immobilized metal affinity chromatography (IMAC). Seed cultures were prepared in 5 mL LB medium using glycerol stocks of *E. coli* BL21 (DE3) cells harboring the constructs pET28a(+)_ADH and pCDFDuet_ADH and supplemented with 40 µg mL^{-1} kanamycin or 50 µg mL^{-1} streptomycin, respectively and grown at 37 °C and 120 rpm overnight. The next day, 1% of the seed cultures were used to inoculate 250 mL LB medium in a 1 L baffled shake flask and the corresponding antibiotic was added. The cultures were grown at 37 °C and 120 rpm to an OD_{600} between 0.6 and 0.8. Recombinant gene expression was induced by adding 0.1 mM isopropyl β-D-1-thiogalactopyranoside (IPTG) performed at 22 °C and 120 rpm for approx. 18 hours. Each culture was then split in half and the cells were harvested via centrifugation at 5,000 x *g* for 25 minutes and 4 °C using JA-10 fixed angle rotor and Beckman Coulter centrifuge. The pellets were resuspended in 15 mL binding buffer B (Table S6), and aminocaproic acid was added as a protease inhibitor at 1 mM final concentration and incubated for ten minutes at room temperature. Sonication was done at 50% duty cycle and output control 5, three times for 1.5 min with a 1-minute break on ice in between cycles using the Sonifier S-250A (Branson Ultrasonics™, USA). From here onward, all steps were done on ice. After breaking the cells, cell-free extract (CFE) was separated from cell debris via centrifugation (5,000 x *g*, 25 min, 4 °C).

The Ni Sepharose 6 Fast Flow purification resin (1.5-2.5 mL resin for 125 mL culture; Cytiva, USA) in a gravity flow column (Econo-Pac® Bio-Rad, USA) was cleaned and calibrated as follows: 2x washing with 20% EtOH, 3x with ddH_2O and 3x with 2 resin volume (RV) of binding buffer B containing 20 mM imidazole. The CFE was loaded onto the calibrated resin and incubated for 15 min on ice.

The flow-through was usually collected with the first washing fraction (FT+W1), using 2x RV of buffer B (Table S6). The column was then washed once more with 2x RV of buffer B (W2) and once with 2x RV of buffer C (W3). The protein of interest was eluted in two to four steps (E1 – E4), in a total volume of 10 mL using buffer D (300 mM imidazole). Afterward, the column was washed 3x each with buffer D, ddH_2O, and 20% EtOH before storing at 4 °C. To remove the imidazole, the elution fractions were dialyzed against buffer A (≥ 500 mL) overnight at 4 °C and slow stirring using Spectra/Por™ 1, 6-8 kDa molecular weight cut-off (MWCO) Standard RC Dry Dialysis tubes (Spectrum™ Labs, USA). After dialysis, the elution fractions were transferred to a Vivaspin® 20, 10 kDa MWCO concentrator spin column (Sartorius, Germany) and washed once with buffer D before finally being concentrated via centrifugation to 2 – 5 mL. The purity of the protein was determined via SDS-PAGE analysis, and the protein concentration was determined using the Pierce™ BCA Protein Assay Kit (Thermo Fisher Scientific, USA) following the MTP procedure as described by the manufacturer. The absorbance at 562 nm was measured with a Synergy™ MX (BioTek Instruments, USA). The expression and purification conditions of the ADHs that were investigated in more detail, namely *Cd*GeDH and HLADH, are summarized in *Table S6*.

The activity of the purified ADHs was determined using **2b**, **3b**, and 1-octanol (positive control) as substrates. The biotransformations were performed in 1.5 mL glass vials in a thermoshaker using 400 rpm and 25 °C. For the reaction, 0.5 mg mL^{-1} ADH, an equimolar amount of *Lp*NOx, and 0.5 mM NAD$^+$ co-factor in a final volume of 1 mL were added. The reactions were initiated by adding 2.5 mM of substrate and performed for 18 h before preparing for analysis via HPLC or GC-FID.

7.3 *Cd*GaDH production

*Cd*GaDH was recombinantly produced using *E. coli* BL21(DE3) as the host strain and IMAC purification analogously to HLADH but using a HEPES-based buffer system instead. Table S6 provides an overview of protein expression conditions and buffers used for purification.

Table S6. Summary of expression and purification conditions used to produce selected ADHs and AlDHs.

Enzyme		*Cd*GeDH	HLADH	*Cd*GaDH
**Plasmid**		pCDFDuet	pET28a(+)	
**Antibiotic**		50 µg mL^{-1} streptomycin	40 µg mL^{-1} kanamycin	
**Expression strain**		*E. coli* BL21 (DE3)		
**Growth conditions**		250 mL LB with antibiotic, 37 °C, 120 rpm to OD_{600} ≈ 0.6 – 0.8		
**Expression conditions**		Induction 0.1 mM IPTG, 22 °C, 120 rpm, approx. 18 h		
**Purification**	**Buffer A**	20 mM NaPi, pH 7.5		50 mM HEPES, pH 7.0; 200 mM NaCl, 10% (v/v) glycerol
	**Buffer B**	20 mM NaPi, pH 7.5; 500 mM NaCl, 20 mM imidazole		50 mM HEPES, pH 7.0; 200 mM NaCl, 10% (v/v) glycerol, 20 mM imidazole
	**Buffer C**	20 mM NaPi, pH 7.5; 500 mM NaCl, 20 mM imidazole		50 mM HEPES, pH 7.0; 200 mM NaCl, 10% (v/v) glycerol, 100 mM imidazole
	**Buffer D**	20 mM NaPi, pH 7.5; 500 mM NaCl, 300 mM imidazole		50 mM HEPES, pH 7.0; 200 mM NaCl, 10% (v/v) glycerol, 300 mM imidazole

7.4 *In vitro* oxidation of 1b by ADH-AlDH cascade

Reaction supernatant of whole-cell biotransformations with *E. coli* BL21 (DE3) pM_AlkB(I238V)-FGTL containing **1b** was prepared as described in section 6. The pH of the supernatant was adjusted to 7.5 with 5 M NaOH. In a tightly sealed 1.5 mL glass vial placed in a 0 °C ice block, purified ADH (*Cd*GeDH or HLADH), *Cd*GaDH, *Lp*NOx (0.5 mg each), and 0.75 mM NAD$^+$ were mixed with 500 µL AlkB reaction supernatant and set to a total reaction volume of 1 mL with 20 mM NaPi, pH 7.5. For time course samples, a master mix was prepared and aliquoted to 1 mL each to avoid evaporation of the volatile reaction intermediates during sampling. The reaction was carried out at 25 °C and 200 rpm in a thermoshaker up to 20.5 h and stopped by adding 100 µL 2 M HCl to 1 mL reaction and 275 µL was directly extracted for GC-analysis (section 8.2). 330 µL of the quenched reaction were subjected to lactonization (section 8.1), before extraction and GC-analysis. The rest of the reaction was stored at -20 °C. For control reactions without **1b**, *E. coli* BL21 (DE3) pCom_AlkL (EVC) reaction supernatant was used. In controls leaving out the other individual reaction components, 20 mM NaPi, pH 7.5 were used instead. Purified *Lp*NOx was obtained from a previously prepared protein stock.[83]

8 Analytics

8.1 Lactonization of 1d to 1e

4-acetoxy-2-methylene butyric acid **1d** was chemically lactonized to **1e** via acidification. Therefore, 300 µL biotransformation samples were transferred to a tightly sealed 1.5 mL glass vial and acidified with 30 µL 2 M HCl. The samples were incubated at 80 °C in a thermoshaker for 1 h and then incubated on ice for 5 min. 275 µL aliquot were then extracted with 250 µL EtOAc and analyzed with GC-MS or GC-FID.

8.2 Analysis by gas chromatography (GC)

For the analysis of whole-cell biotransformation, 250 µL of the samples were quenched with 25 µL of 2 M HCl and extracted with EtOAc containing 1 mM methyl benzoate as ISTD (1:1, v/v) by vigorous shaking for 1 min. Phases were separated by centrifugation (16,000 x *g*, 4 °C, 7 min). The organic phase was dried over $NaSO_4$. 200 µL of the dried extract was directly subjected to GC-analysis.

GC-MS analysis was used to identify and qualitatively confirm the formation of the desired products. The conditions (Table S7) were established using commercially available compounds. The measurements were performed on a Shimadzu GCMS-QP2010 SE instrument equipped with an AOC-20i/s autosampler and injector unit together with a Zebron ZB-5MSi capillary column (30 m × 0.25 mm x 0.25 µm, Phenomenex, USA). Due to a column exchange within the time frame of this study, a slight shift in retention times (Rt) was observed. Rt of **1b** was assigned via purification and NMR-spectroscopy of the formed product. The Rt of the putative aldehyde **1c** was assigned due to its mass spectrogram and elution pattern in comparison with related compounds.

Table S7. Parameters of GC-MS methods (long and short) and retention times of analytes.

**GC parameters**	**Method I (short)**	**Method II (long)**
**Column**	Zebron ZB-5MSi (Phenomenex, 30 m x 0.25 mm x 0.25 µm)	
**Flow Control Mode**	Linear velocity (39.5 cm sec^{-1})	
**Total flow/ Column flow/ Carrier gas**	15 mL min^{-1}/ 1.21 mL min^{-1}/ Helium	
**Injection temperature/ injection volume/ split ratio**	250 °C/ 1 µL/ 9.1	
**Temperature program**	3 min 50 °C, 30 °C min^{-1} to 300 °C, 3 min 300 °C	5 min 50 °C, 40 °C min^{-1} to 300 °C, 5 min 300 °C
**Total program time**	14.33 min	16.25 min
**MS parameters**	**Method I**	**Method II**
**Ion source temperature**	250 °C	
**Interface temperature**	320 °C	
**Mode**	Scan, 30 – 300 m z^{-1}	
	**Retention times**	
**Compounds**	**min (Method I)**	**min (Method II)**
Methyl benzoate (ISTD)	6.69	n. d.
Dicyclopropyl ketone (DCPK; inducer)	5.69	6.97
Isoprenyl acetate **1a**	5.01	6.15
4-acetoxy-2-methylene-butan-1-ol **1b**[n. a]	6.71	8.14
4-acetoxy-2-methylene-butanal **1c**[n. a]	6.39	n. d.
4-acetoxy-2-methylene-butyric acid **1d**	7.52	8.87
Tulipalin A **1e**	5.88	7.15
3-methyl-3,4-epoxy butyl acetate **1f**	6.07	n. d.

n. d.: not determined; n. a.: no authentic standard available

For quantitative analysis, the quenched reaction samples were extracted with EtOAc containing 1 mM of ISTD and then subjected to GC-FID analysis on a Shimadzu Nexis GC-2030 equipped with an AOC-20i Plus autosampler and injector unit and a Zebron ZB-5MSi capillary column (30 m × 0.25 mm x 0.25 µm, Phenomenex, USA). The analytical parameters (Table S8) were established using pure compounds. The concentrations of the analytes were determined by external calibration. To generate the calibration curves, samples with known concentrations of the pure compounds (0 – 6 mM, in duplicates) were extracted from the reaction buffer and treated the same as reaction samples. The concentrations of compounds where no authentic standard was available were calculated based on calibration curves of structurally similar chemicals.

Table S8. Parameters of applied GC-FID method and retention times of analytes. Depending on the substrate used, the according GC-FID method was applied.

Parameters	Method isoprenyl acetate	Method *n*-octane	Method 1-octanol
**Column**	Zebron ZB-5MSi (Phenomenex, 30 m x 0.25 mm x 0.25 µm)		
**Flow Control Mode**	Linear velocity (22 cm sec^{-1})		
**Total flow/ Column flow/ Carrier gas**	15.9 mL min^{-1}/ 1.18 mL min^{-1}/ Nitrogen		
**Injection temperature/ injection volume/ split ratio**	250 °C/ 1 µL/ 10		
**Temperature program**	1 min 50 °C, 20 °C min^{-1} to 250 °C, 2 min 250 °C	1 min 50 °C, 40 °C min^{-1} to 150 °C, 20 °C min^{-1} to 250 °C, 2 min 250 °C	130 °C, 10 °C min^{-1} to 200 C, 50 °C min^{-1} to 250 C, 2 min 250 °C
**FID temperature**	320 °C		
**Total program time**	13.00 min	10.50 min	10.00 min

Retention times

Method isoprenyl acetate		Method *n*-octane		Method 1-octanol	
**Compound**	**min**	**Compound**	**min**	**Compound**	**min**
Methyl benzoate (ISTD)	7.06	Methyl benzoate (ISTD)	5.62	1-decanol (ISTD)	4.74
Dicyclopropyl ketone (DCPK; inducer)	5.12	Dicyclopropyl ketone (DCPK)	4.88	1-octanol	3.56
Isoprenyl acetate **1a**	7.28	*n*-octane	3.94	Octanal	3.33
4-acetoxy-2-methylene-butan-1-ol **1b**[n. a]	6.49	1-octanol	5.41	Octanoic acid	4.0
4-acetoxy-2-methylene-butanal **1c**[n. a]	6.65	Octanal	5.07		
4-acetoxy-2-methylene-butyric acid **1d**	8.14	Octanoic acid	5.92		
Tulipalin A **1e**	8.44				
3-methyl-3,4-epoxy butyl acetate **1f**	6.05				

n. a.: no authentic standard available

8.3 High-performance liquid chromatography (HPLC) analysis

Reactions with **2b** and **3b** were quantitatively analyzed using HPLC-UV. Calibration curves were prepared using commercially available **2b**, **2c**, **2d**, **3b**, and **3d**. Reaction samples and calibration curve samples were prepared equally. To 200 µL sample 1/10 of 2 M HCl and 250 µL methanol were added to quench the reaction and mixed vigorously for 1 min. Then, the samples were centrifuged for 45 min at maximum speed, and 250 µL of each sample was transferred to a glass vial. The samples were analyzed on a Shimadzu LC-MS 2020 system equipped with a UV detector and a Phenomenex C18 column (USA). Calibration curves were prepared from three stocks and measured in triplicates in a concentration range of 0 – 10 mM via dilutions with reaction buffer (20 mM NaPi, pH 7.5). Since the aldehyde **3c** was not commercially available, it was only analyzed semi-quantitatively by comparing peak areas. The Rt of **3c** was determined based on the elution pattern of **2b**-**d**, where aldehyde elutes at last, and comparison to control reactions. Table S9 summarizes the HPLC method parameters, retention times of the analytes, and the wavelengths at which they were quantified.

Table S9. HPLC parameters of the method used for quantitative analysis of reactions with **2b** and **3b** and retention times and detection wavelength of each compound.

**Column**	Phenomenex C18 HPLC column	**Compound**	**Rt (min)**	**Detection wavelength (nm)**
**Eluent**	0.1% HCOOH aq./CH_3CN	2-methallyl alcohol **2b**	1.34	200
**Ratio**	1:25	Methacrolein **2c**	1.98	210
**Flow rate**	1.0 mL min^{-1}	Methacrylic acid **2d**	1.55	200
**Detector**	PDA (UV)	2-ethylallyl alcohol **3b**	1.98	200
**Column temperature**	40 °C	2-ethylacrolein **3c**[n. a]	2.68	200
**Run time**	4.21 min	2-ethylacrylic acid **3d**	2.16	200
**Injection volume**	10 µL			

n. a.: no authentic standard available

8.4 NMR-spectroscopy

^1H- and ^{13}C-spectra were recorded using an Avance™ III 300 MHz FT NMR spectrometer. For the analysis, 5-10 mg of analyte were dissolved in $CDCl_3$. The spectroscopic data was compared to reported and predicted spectra.

9 Synthesis of 1f

The epoxide 3-methyl-3,4-epoxybutyl acetate **1f** was expected to be a potential product of isoprenyl acetate **1a** conversions with oxygenases. Hence, a standard of **1f** was synthesized via acetylation of 2-(2-methyl-2-oxiranyl)ethanol **5f** using a commercially available lipase from *Pseudomonas cepacia*. GC-MS analysis of the lipase-catalyzed reaction showed a product peak at 6.07 min (Figure S2) that was confirmed as **1f** via NMR-spectroscopy. The recorded spectra agreed with the literature and predicted data.[31]

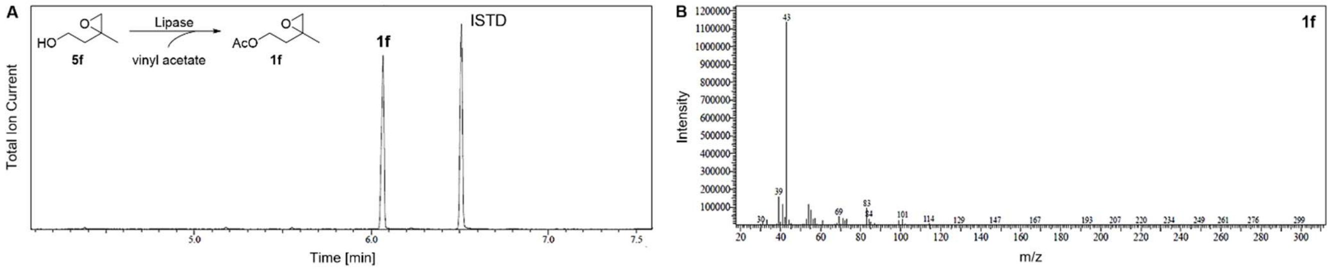

Figure S2. (A) GC-MS chromatogram of lipase-catalyzed acetylation of 2-(2-Methyl-2-oxiranyl)ethanol **5f** to 3-methyl-3,4-epoxybutyl acetate **1f** (Rt: 6.07 min) and (B) mass spectrogram of **1f**.

10 UPO-catalyzed transformation of isoprenyl acetate

Unspecific peroxygenases were considered promising candidates for catalyzing the terminal hydroxylation of isoprenyl acetate **1a** to **1b**, the key reaction in the artificial route towards tulipalin A (Scheme 1B). In a screening of 77 different UPOs from Aminoverse (Nuth, The Netherlands) we found that 72 UPOs, including *Aae*UPO, are active towards **1a.** GC-MS analysis (Figure S3A-B) showed a product peak (Rt: 6.07 min) with a mass spectrum that did not correspond to the desired alcohol **1b** but rather to the epoxide **1f** instead. This was confirmed by comparison with the product **1f** obtained via acetylation of **5f** (Figure S1, Figure S3C). In addition, the NMR spectrum of the isolated and purified UPO-product (^1H NMR, $CDCl_3$: *δ* 1.29 (s, 3H), *δ* 1.74-1.87 (m, 2H), *δ* 2.34 (s, 3H), *δ* 2.53 (d, 1H, *J* = 4.52), *δ* 2.58 (d, 1H, *J* = 4.56), *δ* 4.01-4.20 (m, 2H)) was in agreement with the spectrum of **1f** synthesized by acetylation of **5f**.

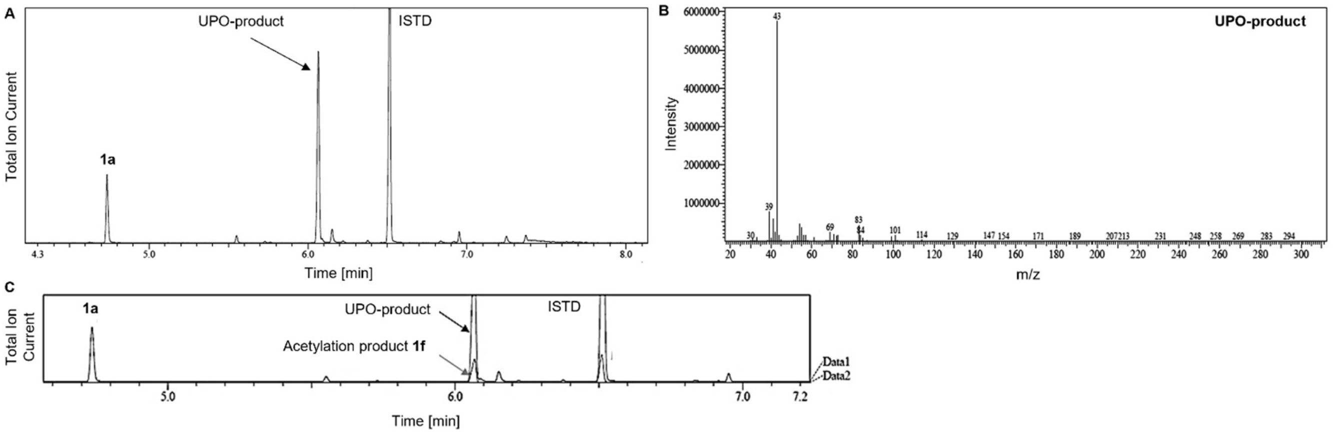

Figure S3. (A) GC-MS chromatogram of *Aea*UPO-mediated conversion of isoprenyl acetate **1a** gave a prominent product peak at 6.07 min. (B) Mass spectrogram of the UPO-product. (C) Comparison of the chromatogram of the UPO reaction with the standard **1f** produced by lipase-catalyzed acetylation of **5f**.

Table S10 provides a semi-qualitative summary of **1f** formation from **1a** by the 77 UPOs from the enzyme panel. All UPOs, except for UPO 27, UPO 30, UPO 41, UPO 43, and UPO 48 converted **1a**, with *Aae*UPO yielding the highest amount of **1f**.

Table S10. Out of the 77 UPOs (Aminoverse, Nuth, The Netherlands), 72 UPOs converted **1a** to the epoxide **1f,** while none gave the desired terminal alcohol **1b**. +++, ++, and + indicate GC-MS peak areas of **1f** (magnitude of ≥ 10^7, ≥ 10^6, ≥ 10^5, respectively) while – indicates no product formation (*n=1*).

UPO1<br>++	UPO5<br>+++	UPO3<br>+++	UPO21<br>++	UPO29<br>++	UPO42<br>++	*Aae*UPO*<br>+++	UPO56<br>+++	UPO13M2<br>+++	UPO36M1<br>++
UPO4<br>+++	UPO8<br>+++	UPO28<br>+	UPO24<br>+++	UPO32<br>+++	UPO38<br>+++	UPO49<br>+++	UPO57<br>+++	UPO13M5<br>+++	UPO36M2<br>+++
UPO7<br>+	UPO11<br>+++	UPO31<br>++	UPO19<br>+++	UPO26<br>+	UPO44<br>++	UPO50<br>+++	UPO58<br>+++	UPO13M4<br>+++	UPO36M3<br>+++
UPO10<br>++	UPO14<br>++	UPO6<br>+	UPO34<br>++	UPO45<br>++	UPO27<br>-	UPO51<br>++	UPO59<br>++	UPO13M7<br>++	UPO36M4<br>++
UPO13<br>++	UPO17<br>++	UPO9<br>+	UPO37<br>++	UPO46<br>+	UPO48<br>-	UPO52<br>++	UPO60<br>++	UPO13M8<br>++	UPO36M5<br>++
UPO16<br>++	UPO20<br>++	UPO12<br>++	UPO39<br>+	UPO35<br>++	UPO30<br>-	UPO53<br>++	UPO61<br>++	UPO13M9<br>++	neg. control<br>-
UPO22<br>++	UPO25<br>++	UPO15<br>++	UPO41<br>-	UPO47<br>++	UPO33<br>++	UPO54<br>++	UPO13M3<br>++	UPO13M10<br>++	UPO13<br>++
UPO2<br>++	UPO23<br>+	UPO18<br>++	UPO43<br>-	UPO40<br>+	UPO36<br>++	UPO55<br>++	UPO13M1<br>++	UPO12M11<br>++	

11 alkBFGT*(HJL)* expression and whole-cell biotransformations

Polycistronic expression of the *alk*-operon was confirmed by SDS-PAGE. An example of the expression of *PpGPo1alkBFGTHJL* expression is given in Figure S4. AlkB, the outer-membrane transporter AlkL, and the membrane-anchored AlkJ were expected in the insoluble fraction. The soluble redox-system AlkFGT and AlkH in the soluble fraction.

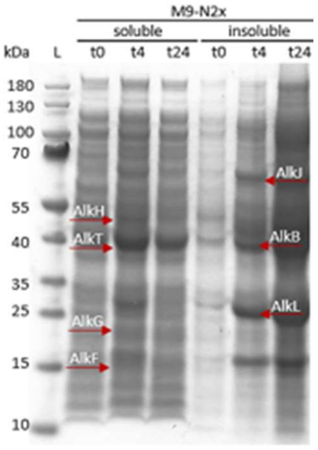

Figure S4. SDS-PAGE analysis of the soluble (s) and insoluble fraction of *alkBFGTHJL* expression. The membrane (m) proteins are expected in the insoluble fraction. AlkB: 46 kDa (m), AlkF: 15 kDa (s), AlkG: 19 kDa (s), AlkS: 99 kDa (s), AlkT: 41 kDa (s), AlkL: 25 kDa (m), AlkJ: 61 kDa (m), AlkH: 53 kDa (s).

Whole-cell biotransformations using *E. coli* BL21(DE3) p*alkB(mut/homolog)FGT(L)* and **1a** target or *n*-octane as reference substrates were qualitatively analyzed by GC-MS and quantitatively by GC-FID. Initial biotransformation with cells expressing *PpGPo1alkBFGT* and **1a** as substrate showed the formation of a product peak at 6.71 min with a mass spectrogram fitting to the desired terminal alcohol **1b** (Figure S5). To confirm the formation of 4-acetoxy-2-methylene-butan-1-ol **1b** by AlkB, the product was isolated and purified from whole-cell reactions and analyzed via NMR-spectroscopy (^{1}H NMR ((300 MHz, $CDCl_3$), δ 5.12 (s, 1H), 4.94 (s, 1H), 4.22 (t, *J* = 6.72 Hz, 2H), 4.11 (s, 2H), 2.42 (t, *J* = 6.39 Hz, 2H), 2.05 (s, 3H) ppm). The recorded spectrum was in agreement with the literature.[46]

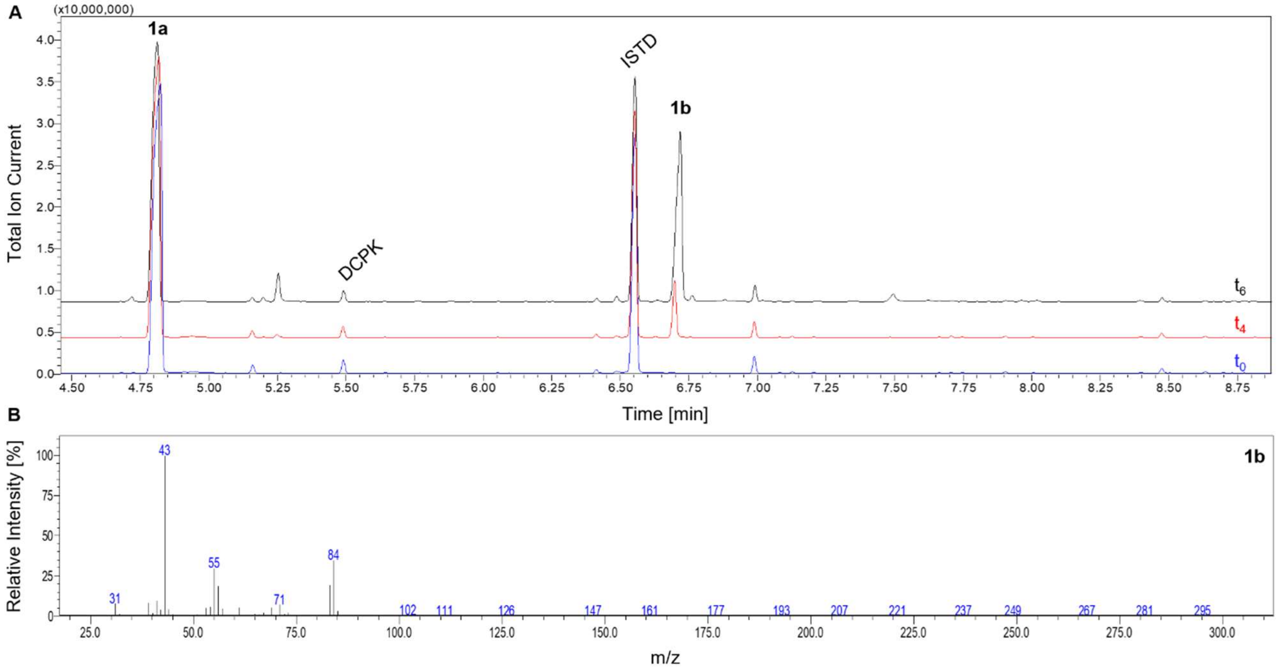

Figure S5. (A) GC-MS analysis of whole-cell biotransformations with cells producing PpGPo1AlkBFGT and **1a** as substrate (Rt: 4.82 min) shows a peak appearing after 4h at 6.71 min with a mass spectrum (B) fitting to the desired product **1b**.

As the PpGPo1AlkBFGT system could selectively hydroxylate **1a** to **1b**, we were interested if we could further oxidize **1b** in an *in vivo* cascade to **1c** and **1d** by expanding the *alk*-operon with the ADH (AlkJ) and AlDH (AlkH) from *P. putida* GPo1. However, GC-MS analysis of whole-cell biotransformations with cells harboring p*PpGpo1alkB-FGTHJ* and **1a** as substrate did show the formation of **1b**, but no peaks that could be assigned to aldehyde **1c** or the carboxylic acid **1d** (Figure S6). Reaction with cells co-expressing the transporter *alkL*, showed similar results with only **1b** formation (data not shown). We assume that AlkJ is not active towards **1b** representing the bottleneck in the oxidation. Hence, we screened a set of 20 ADHs with β-substituted alcohols similar to **1b** (section 12).

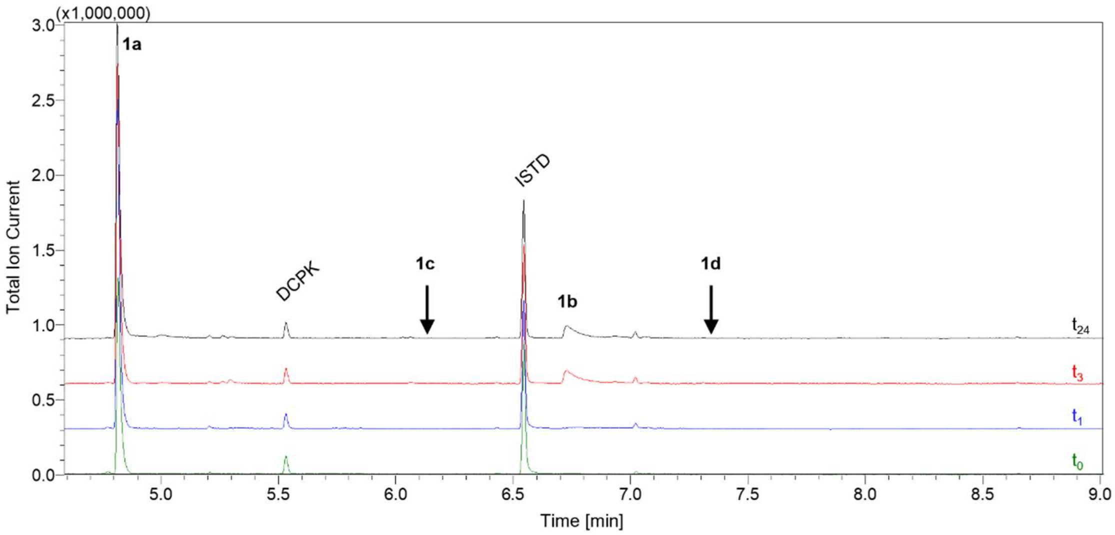

Figure S6. GC-MS analysis of whole-cell biotransformation of **1a** with cells expressing the operon *PpGpo1alkB-FGTHJ* yielded the terminal alcohol **1b** but no oxidation to the aldehyde **1c** or the carboxylic acid **1d** was observed.

It was assumed that other alkane MOs related to AlkB also selectively hydroxylate **1a** and some might exhibit even higher activities than AlkB from *P. putida* GPo1. This was exemplified by testing four homologous alkane MOs from *Pseudomonas putida* P1 (PpP1AlkB), *Marinobacter* sp. (M_AlkB), *Alkanivorax borkumensis* (AboAlkB) and *Acetionobacter baylyi* (AbaAlkB). An alignment of the respective protein sequences is shown in Figure S7.

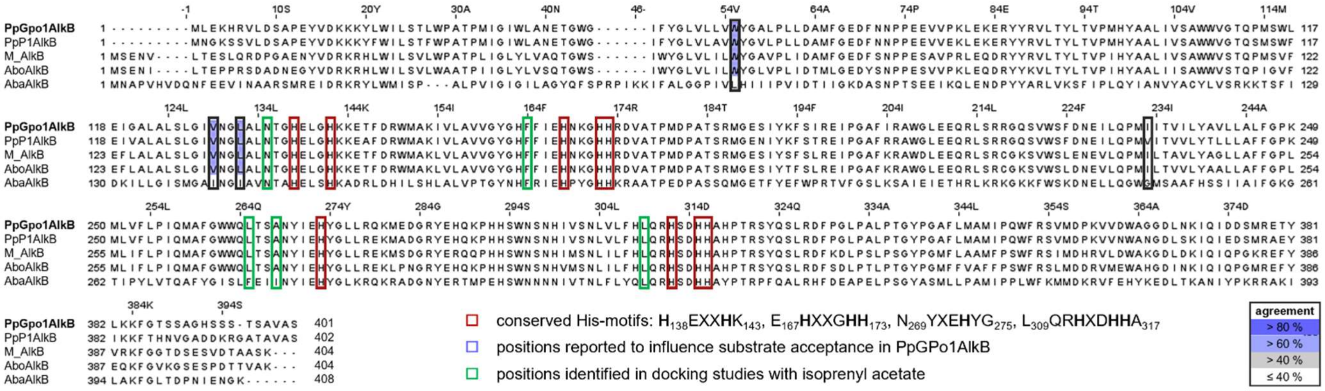

Figure S7. Protein sequence alignment of selected AlkB-homologs. The nine conserved histidines that coordinate the iron atoms in the catalytic center are marked with red boxes. Residues reported to influence the substrate spectrum of AlkB from *P. putida* GPo1 are highlighted in blue. Positions identified in this study by docking of isoprenyl acetate **1a** in PpGPo1AlkB are framed green.[47,48]

The different AlkB homologs and variants were tested in whole-cell biotransformations, and the enzymatic activities (U g_{cdw}^{-1}) for the two substrates isoprenyl acetate (Figure 2A) and *n*-octane (Figure S8B) were determined from biological triplicates and calculated as µmol of product formed per min and g_{cdw}^{-1}. AbaAlkB was poorly produced and hence not used for whole-cell biotransformations.

The presence of AlkL in whole-cell biotransformations with the reference substrate *n*-octane (Figure S8A) shows similar trends as to **1a** (Figure 2A) for all four homologs, with M_AlkB being the most active. Comparing the activity of the rationally designed PpGPo1AlkB variants with **1a** (Figure 2B) and *n*-octane (Figure S8B) shows that I233V appears to be an activity driver for both substrates. The variant F164L, identified via docking with **1a**, showed higher activity towards the target substrate **1a** while being less active towards the natural substrate. The combination of the mutations F164L and I233V with the co-expression of *alkL* are shown in Figure S8C. The effect of the homologous mutations in M_AlkB (F169L and I238L) are presented in Figure S8D. Interestingly, mutations of the residue F164 (F169 in M_AlkB) seem to yield higher amounts of the overoxidation products octanal and octanoic acid. Some overoxidation by *E. coli* native is expected, but it might also be attributed to AlkB's ability to overoxidize its substrates to aldehydes, which has been reported previously.[42,84,85]

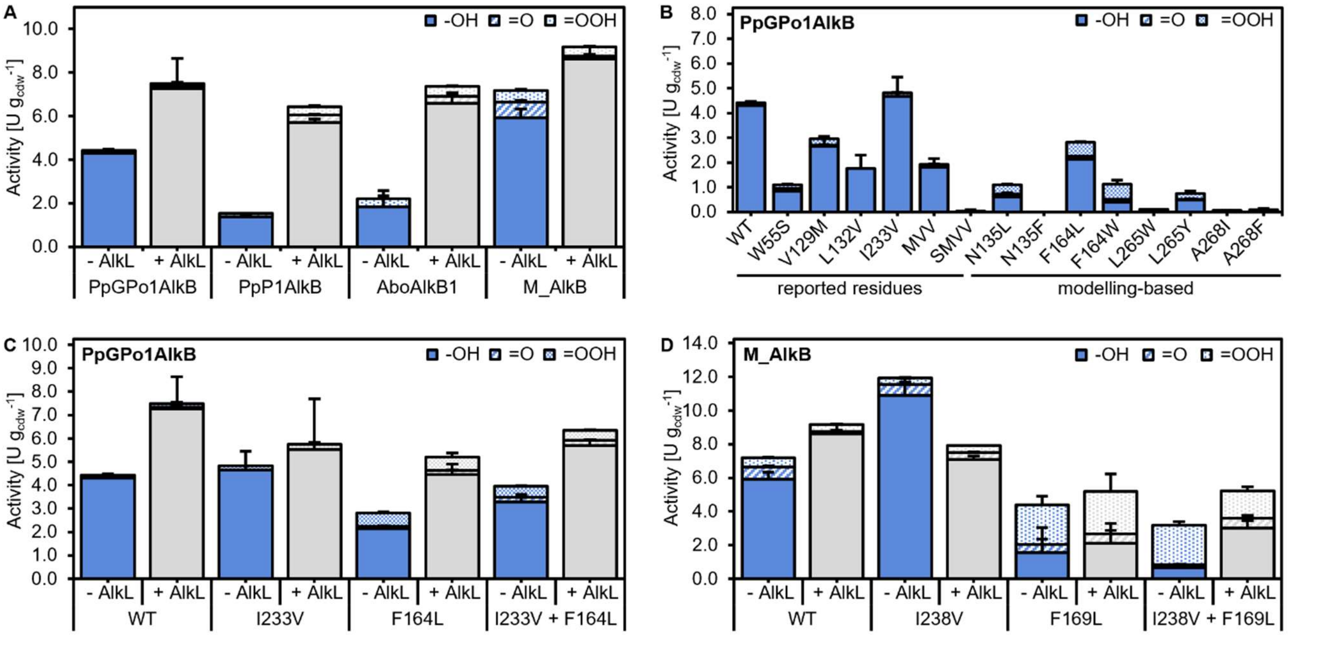

Figure S8. Formation of 1-octanol (-OH; filled), octanal (=O; striped), and octanoic acid (=OOH; dotted) by AlkB homologs and variants in whole-cell biotransformations with *n*-octane. (A) The presence of transporter AlkL (light grey) led to higher activities for all four tested AlkB-homologs compared to reactions without the transporter (light blue). (B) Screening of the rationally designed PpGPo1AlkB variants with *n*-octane (data without AlkL). (C) and (D) show activities of selected PpGPo1AlkB and M_AlkB variants, respectively, considering the transporter's effect and the double-mutation. The data is represented as arithmetic mean and standard deviation indicated by error bars (*n=3*).

The activities towards isoprenyl acetate **1a** and *n*-octane of all AlkB homologs and variants tested in this study are summarized in Table S11 (data without transporter AlkL). The preference P of the enzyme is given as the ratio of the activity towards **1a** over *n*-octane, while the preference shift P_V/P_{WT} indicates the change in substrate preference caused by the mutation.

Table S11. Specific activities of AlkB wildtypes (WT) and variants (V) without AlkL present. The substrate preference P indicates the activity of isoprenyl acetate **1a** over *n*-octane, while the preference shift P_V/P_{WT} compares the change of substrate preference of V over the WT. Activity with **1a** is given as µmol **1b** formed per min and g_{cdw}. Activity with *n*-octane is given as the sum of µmol 1-octanol, octanal, and octanoic acid formed per min and g_{cdw}. The data is represented as arithmetic mean ± standard deviation (*n=3*).

Enzyme	Variant (V)	Activity [U g_{cdw}^{-1}]		Preference P	Preference shift P_V/P_{WT}
		Isoprenyl acetate	*n*-octane		
PpGPo1AlkB	WT	0.28 ± 0.04	4.43 ± 0.16	0.1	1.0
PpGPo1AlkB	W55S[47]	0.04 ± 0.00	1.11 ± 0.10	0.0	0.5
PpGPo1AlkB	V129M[48]	0.00 ± 0.00	2.95 ± 0.45	0.0	0.0
PpGPo1AlkB	L132V[48]	0.00 ± 0.00	1.77 ± 0.54	0.0	0.0
PpGPo1AlkB	I233V[48]	0.74 ± 0.06	4.83 ± 0.79	0.2	2.4
PpGPo1AlkB	MVV[48]	0.00 ± 0.00	1.92 ± 0.37	0.0	0.0
PpGPo1AlkB	SMVV[47,48]	0.00 ± 0.00	0.05 ± 0.04	0.0	0.0
PpGPo1AlkB	N135L	0.00 ± 0.00	1.11 ± 0.04	0.0	0.0
PpGPo1AlkB	N135F	0.00 ± 0.00	0.00 ± 0.00	-	-
PpGPo1AlkB	F164L	0.82 ± 0.06	2.81 ± 0.08	0.3	4.6
PpGPo1AlkB	F164W	0.38 ± 0.06	1.12 ± 0.24	0.3	5.4
PpGPo1AlkB	L265W	0.00 ± 0.00	0.11 ± 0.00	0.0	0.0
PpGPo1AlkB	L265Y	0.10 ± 0.01	0.75 ± 0.16	0.1	2.1
PpGPo1AlkB	A268I	0.00 ± 0.00	0.07 ± 0.01	0.0	0.0
PpGPo1AlkB	A268F	0.08 ± 0.01	0.08 ± 0.04	1.0	15.1
PpGPo1AlkB	I233V+F164L	0.75 ± 0.14	3.95 ± 0.39	0.2	3.5
M_AlkB	WT	0.64 ± 0.06	7.18 ± 0.35	0.1	1.0
M_AlkB	I238V	1.70 ± 0.05	11.92 ±1.14	0.1	1.6
M_AlkB	F169L	1.02 ± 0.06	4.38 ± 1.29	0.3	3.2
M_AlkB	I238V+F169L	0.59 ± 0.10	3.18 ± 0.36	0.2	2.5
PpP1AlkB	WT	0.48 ± 0.05	1.54 ± 0.01	0.3	-
AboAlkB	WT	0.41 ± 0.04	2.22 ± 0.87	0.2	-

11.1 Control reaction with isoprenol 5a

Conversion of **5a** to 2-methylene-1,4-butandiol **5b** by AlkBFGT was also investigated to see if there would be any potential cross-reactions in whole-cell applications with *in vivo* acetylation of **5a** to isoprenyl acetate **1a**. The reaction was performed analogously to the small-scale biotransformations with **1a** and *n*-octane. 5 mM of **5a** was used as the initial substrate concentration and incubated for up to 48 h before extraction with EtOAc (1:1). The dried organic phase was derivatized by *N,O*-Bis(trimethylsilyl)trifluoroacetamide (BSTFA). Peaks were assigned by comparison to commercially available standards. The silylated substrate **5a** elutes at 6.05 min. The mono-silylated **5b** derivates elute at around 8 min and the di-silylated **5b** at 8.54 min. The reaction samples do not show any peaks which could be assigned to 2-methylene-1,4-butandiol indicating that **5a** is not accepted as substrate by AlkB. Hence, a cross-reaction with the acetylation of **1a** in a whole-cell procedure is not expected (Figure S9).

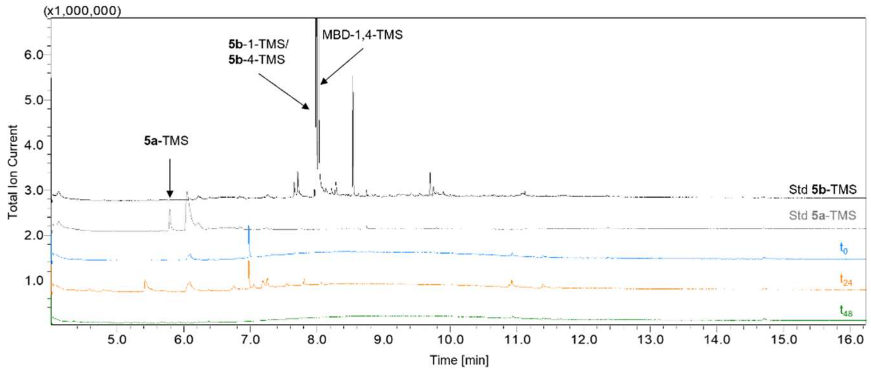

Figure S9. Conversion of isoprenol **5a** by PpGPo1AlkBFGT. Reaction samples were taken directly at the start of the reaction (blue), after 24 h (orange) and 48 h (green), and derivatized using BSTFA prior to GC-MS analysis. The reaction samples were compared to the derivatized standards of **5a** (grey) and **5b** (black) showing that **5a** is not converted to **5b** by AlkB. Retention times of analytes: **5a**-TMS: 6.05 min; **5b**-1-TMS: 7.99 min; **5b**-4-TMS: 8.02 min; **5b**-1,4-TMS: 8.54 min.

12 ADH screening

The initial whole-cell screening of the ADH panel listed in Table S3 with **2b**, **3b**, and 1-octanol is shown in Figure S10. The five enzymes that showed promising results in the screening were investigated more closely. Firstly, *Tc*ADH1 and HLADH were picked due to their high preference towards 1-octanol (positive control) and converted 23% and 18% to the corresponding aldehyde, respectively. For both enzymes, octanoic acid formation was observed as well. *Tg*Ghbd1 produced mostly octanoic acid and only small amounts of octanal. In reactions with *Cd*GeDH, **2c** and **2d** were formed from **2b,** and traces of **3d** were detected when **3b** was used as substrate. Furthermore, *Pp*BDH yielded small amounts of **2d**. Interestingly, the aldehyde **3c** was not detected in the screening with any of the enzymes when **3b** was used as a substrate.

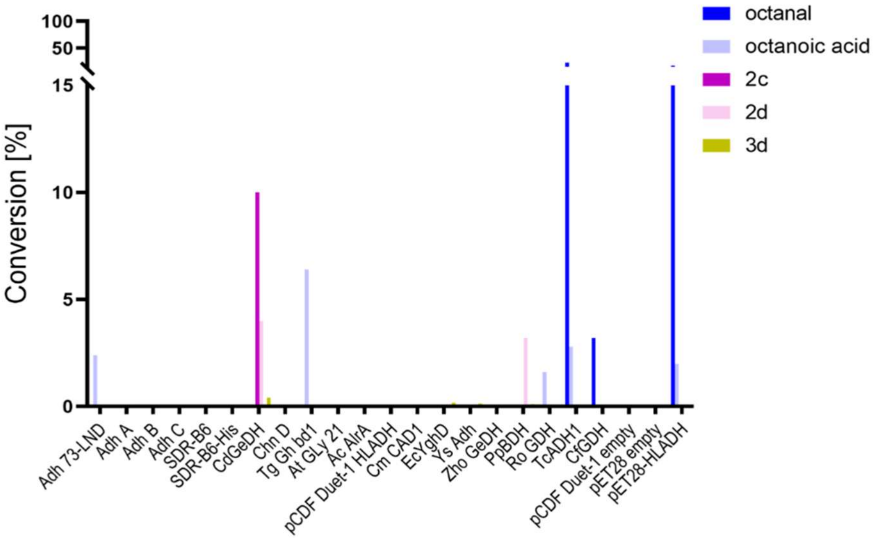

Figure S10. Conversion of **2b**, **3b**, and 1-octanol to aldehyde and carboxylic acid in a whole-cell screening of the ADH-panel. 1-octanol conversions were analyzed via GC-FID and **2b** and **3b** conversions via UHPLC-UV. The formation of **3c** was not observed at all.

13 Shake flask production of ADHs and AlDHs

ADHs that showed promising results in the whole-cell screening with the tested substrates (1-octanol, **2b**, and **3b)**, namely *Cd*GeDH, *Tg*Ghbd1, *Pp*BDH, *Tc*ADH1, and HLADH were subjected to shake flask expression and purification. Out of those five enzymes, we could express and purify HLADH and *Cd*GeDH in their active form (Figure S11A and B, respectively). Those two enzymes showed activity toward the test substrates **2b** and **3b** in their purified form. His-tag purification of *Tc*ADH1 and *Pp*BDH was unsuccessful and purified *Tg*Ghbd1 was not active towards the test substrates. Thus, only *Cd*GeDH and HLADH were further investigated.

Purified geranial dehydrogenase *Cd*GaDH was used to oxidize **1c**. SDS-PAGE analysis of His-tag purification of *Cd*GaDH is shown in Figure S11C with the enzyme appearing at ~58 kDa.

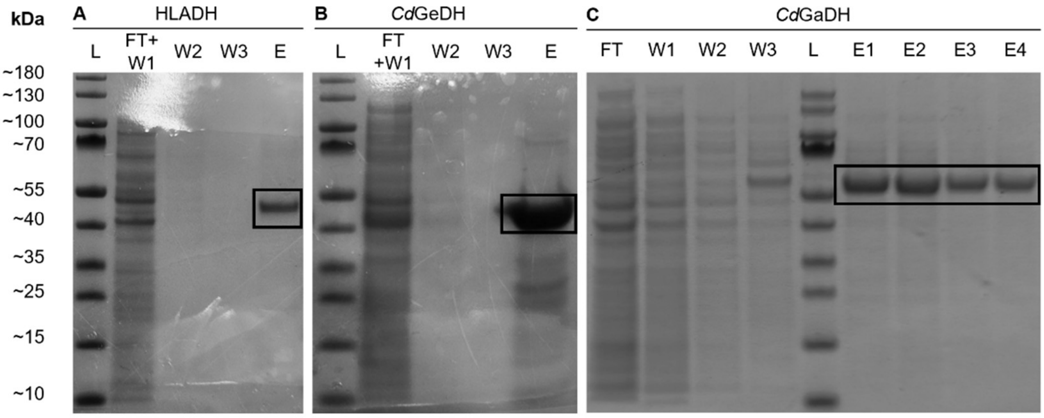

Figure S11. Purification of (A) HLADH, (B) *Cd*GDH, and (C) *Cd*GaDH with expected sizes of ~ 40 kDa, ~39 kDa, and ~53 kDa, respectively. The gel shows the flowthrough (FT) of the cell-free extract, washing fractions (W), and elution fractions (E) of the protein of interest. The sizes of the protein were compared to the PageRuler™ Prestained Protein Ladder (L; 10 – 180 kDa) from Thermo Fisher Scientific.

14 2b and 3b conversion by HLADH and *Cd*GeDH

In vitro biocatalysis was done using purified HLADH and CdGeDH with **2b** and **3b** as test substrates. Reactions with and without NAD^+ regeneration by *Lp*NOx were compared, clearly showing its effect. Figure S12 and Figure S13 show the HPLC data obtained after 18 h reaction with HLADH and *Cd*GeDH, respectively. When using the *Lp*NOx, HLADH resulted in ~50% conversion of **2b** to **2c**, and ~92% conversion of **3b** to **3c**. Using HLADH we could also observe up to ~60% conversion to the acid **3d** when **3b** was used as a substrate, but this was inconsistent and showed batch-to-batch variation (Figure S12C). At the same time, *Cd*GeDH and *Lp*NOx resulted in ~84% conversion of **2b** to **2c**, and ~36% conversion of **3b** to **3c**.

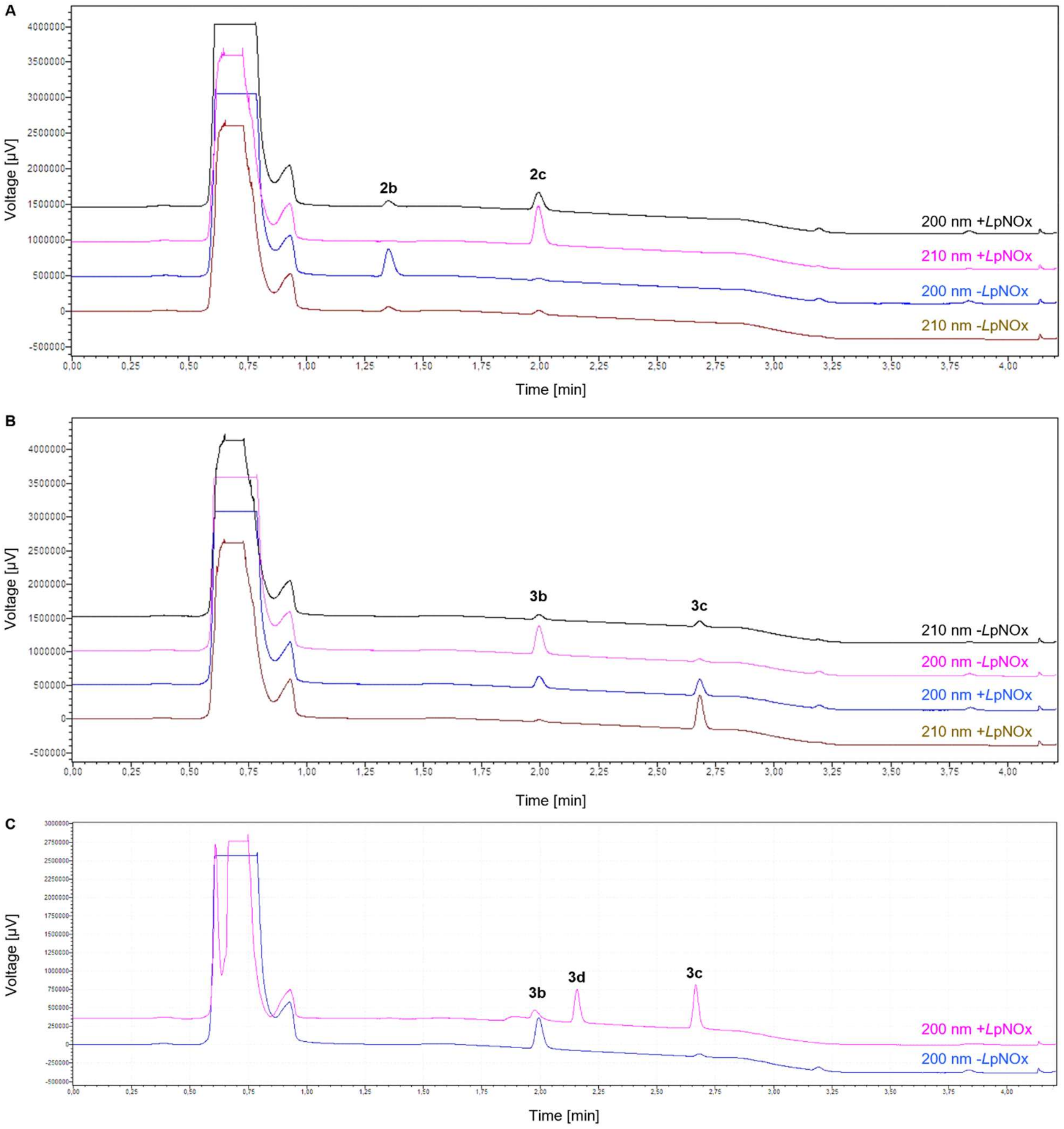

Figure S12. HPLC analysis at 200 and 210 nm of 18 h reaction samples with HLADH and (A) **2b** and (B) **3b** as substrates. NAD^+ was regenerated by *Lp*NOx (+) and compared to reactions without co-factor recycling (-). (C) In some batches, partial overoxidation to the acid **3d** was observed when using HLADH together with *Lp*NOx and **3b** as substrate.

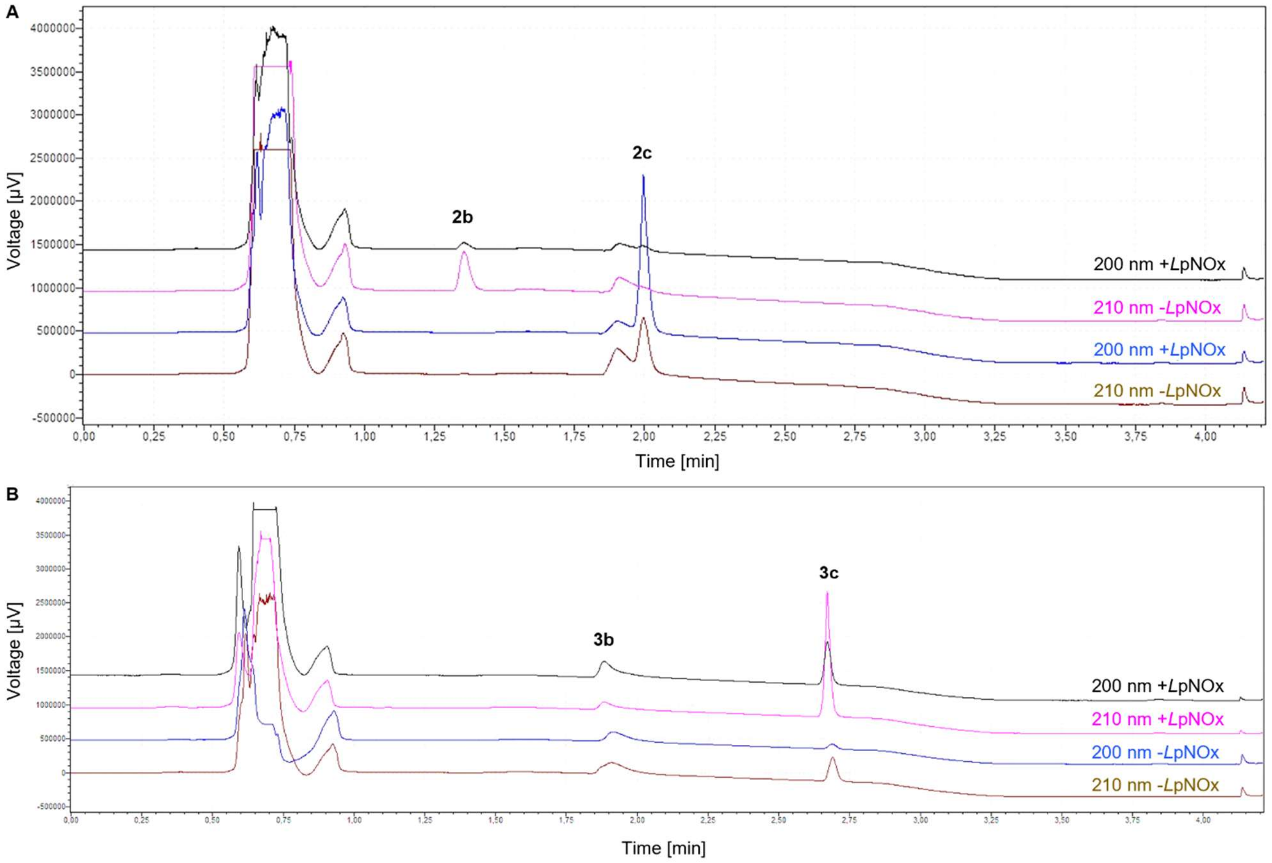

Figure S13. HPLC analysis at 200 and 210 nm of 18 h reaction samples with *Cd*GeDH and (A) **2b** and (B) **3b** as substrates. NAD^+ was regenerated by *Lp*NOx (+) and compared to reactions without co-factor recycling (-).

15 1b **oxidation by ADH-AlDH cascade and lactonization to** 1e

In an *in vitro* cascade employing ADH and AlDH together with *Lp*NOx for NAD^+ recycling, we could convert **1b** to **1d**. Acidification with HCl and incubation at 80 °C yielded the desired lactone **1e**. **1b** was added as supernatant from whole-cell hydroxylation reactions with **1a**. Figure S14 shows qualitative GC-MS analysis of 18 h samples directly extracted (A) or lactonized (B) from the whole cascade with *Cd*GeDH, *Cd*GaDH, and *Lp*NOx as well as control reactions. Figure S15 shows the cascade reactions with HLADH, *Cd*GaDH, and *Lp*NOx. Only in reactions with ADH and AlDH present **1d** was formed. In reactions without an ADH or without NAD^+ **1b** was not oxidized, and in case no AlDH was present the reaction stopped at the aldehyde **1c**. Less **1d** was formed without co-factor recycling by *Lp*NOx.

In quantitative analysis via GC-FID of independent biological replicates (*n*=2), we could confirm ~95% conversion of the initially applied 0.58 mM **1b** to **1d** after 20.5 h by the enzyme cascade consisting of *Cd*GeDH, *Cd*GaDH, and *Lp*NOx. Upon lactonization, 83% overall conversion of **1b** to tulipalin A was observed.

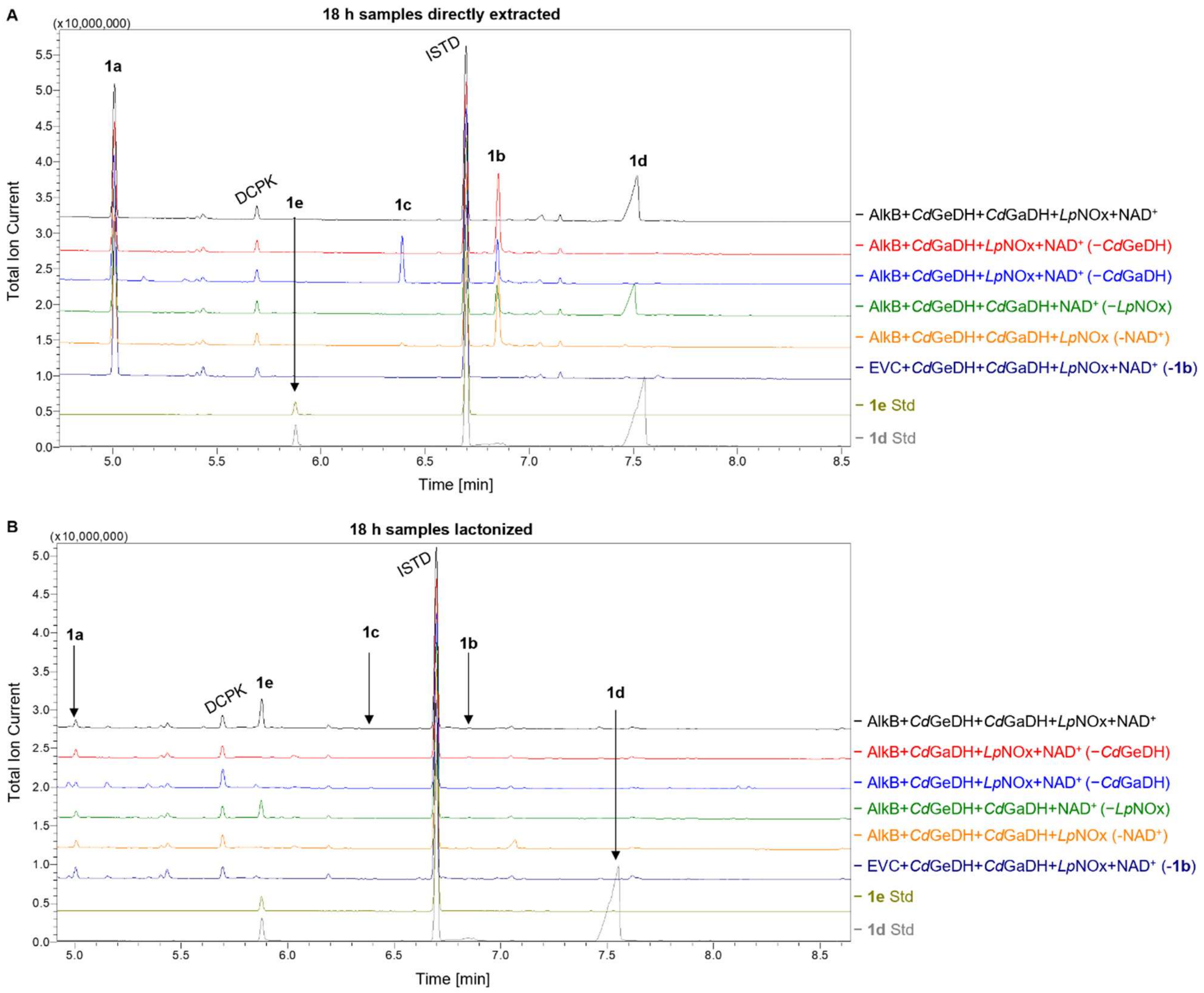

Figure S14. (A) GC-MS analysis of 18 h samples of *in vitro* oxidation of **1b** to **1d** by *Cd*GeDH and *Cd*GaDH and (B) lactonization to **1e**. Whole-cell reaction supernatant of **1a** conversions to **1b** by M_AlkB(I238V)FGTL was used as substrate and *Lp*NOx was added to recycle NADH back to NAD^+. In control reactions, the individual components were substituted with reaction buffer, and for controls without **1b** supernatant of EVC reactions was used instead of M_AlkB(I233V)FGTL.

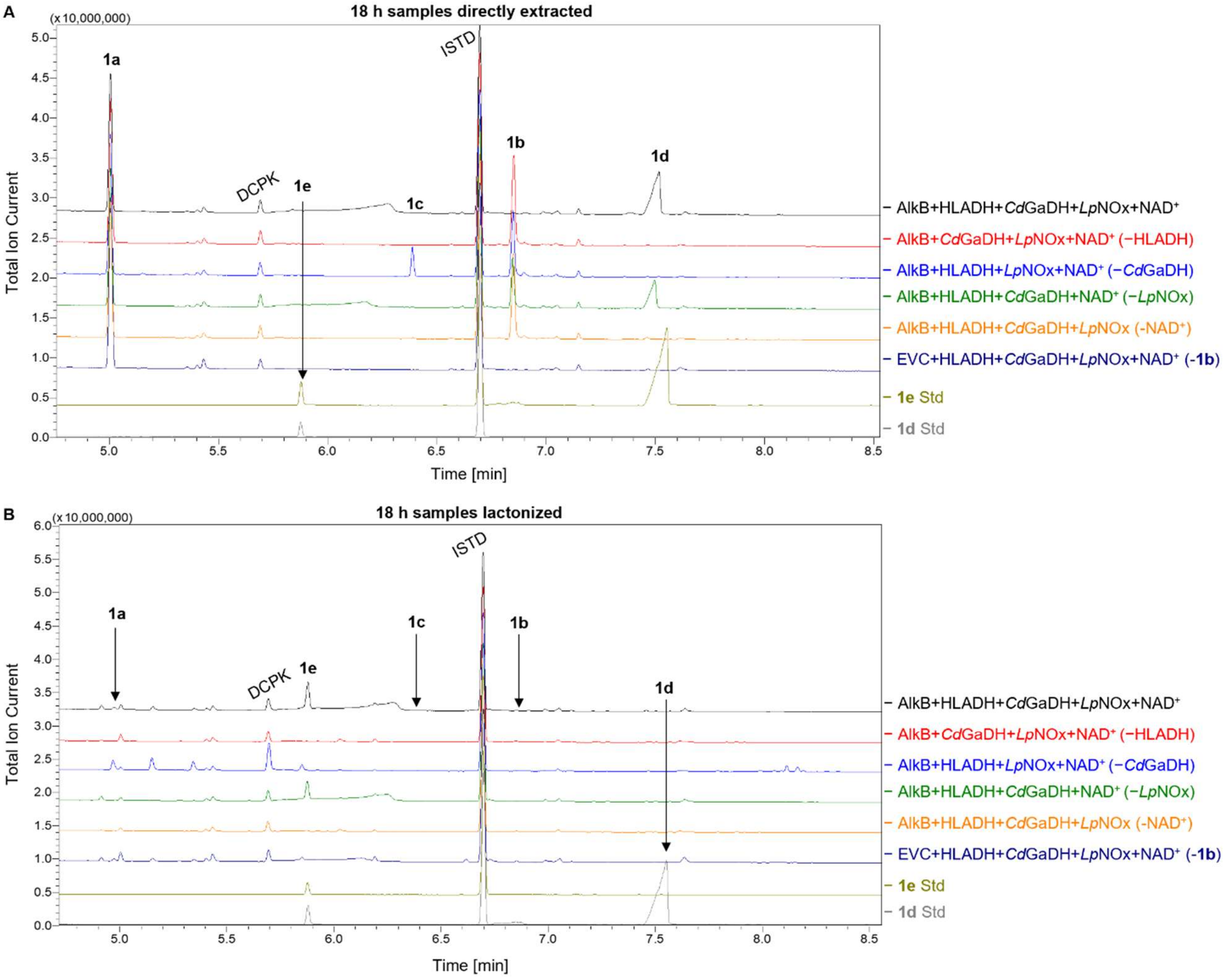

Figure S15. (A) GC-MS analysis of 18 h samples of *in vitro* oxidation of **1b** to **1d** by HLADH and *Cd*GaDH and (B) lactonization to **1e**. Whole-cell reaction supernatant of **1a** conversions to **1b** by M_AlkB(I238V)FGTL was used as substrate and *Lp*NOx was added to recycle NADH back to NAD^+. In control reactions, the individual components were substituted with reaction buffer, and for controls without **1b** supernatant of EVC reactions was used instead of M_AlkB(I233V)FGTL.
